# Resting Energy Expenditure and Body Composition in Children and Adolescents With Genetic, Hypothalamic, Medication-Induced or Multifactorial Severe Obesity

**DOI:** 10.3389/fendo.2022.862817

**Published:** 2022-07-11

**Authors:** Ozair Abawi, Emma C. Koster, Mila S. Welling, Sanne C.M. Boeters, Elisabeth F. C. van Rossum, Mieke M. van Haelst, Bibian van der Voorn, Cornelis J. de Groot, Erica L. T. van den Akker

**Affiliations:** ^1^Dept. of Pediatrics, div. of Endocrinology, Erasmus MC-Sophia Children’s Hospital, University Medical Center Rotterdam, Rotterdam, Netherlands; ^2^Obesity Center CGG, Erasmus MC, University Medical Center Rotterdam, Rotterdam, Netherlands; ^3^Dept. of Dietetics, Erasmus MC, University Medical Center Rotterdam, Rotterdam, Netherlands; ^4^Dept. of Internal Medicine, div. of Endocrinology, Erasmus MC, University Medical Center Rotterdam, Rotterdam, Netherlands; ^5^Dept. of Human Genetics, Amsterdam University Medical Center, Location AMC, University of Amsterdam & Location VUmc, Vrije Universiteit Amsterdam, Amsterdam, Netherlands; ^6^Dept. of Pediatrics, Willem-Alexander Children’s Hospital, Leiden University Medical Center, Leiden, Netherlands

**Keywords:** childhood obesity, metabolism, metabolic rate, monogenic obesity, syndromic obesity, PHP1a, Temple syndrome, 16p11.2 deletion syndrome

## Abstract

**Background:**

Pediatric obesity is a multifactorial disease which can be caused by underlying medical disorders arising from disruptions in the hypothalamic leptin-melanocortin pathway, which regulates satiety and energy expenditure.

**Aim:**

To investigate and compare resting energy expenditure (REE) and body composition characteristics of children and adolescents with severe obesity with or without underlying medical causes.

**Methods:**

This prospective observational study included pediatric patients who underwent an extensive diagnostic workup in our academic centre that evaluated endocrine, non-syndromic and syndromic genetic, hypothalamic, and medication-induced causes of obesity. REE was assessed by indirect calorimetry; body composition by air displacement plethysmography. The ratio between measured REE (mREE) and predicted REE (Schofield equations), REE%, was calculated, with decreased mREE defined as REE% ≤90% and elevated mREE ≥110%. Additionally, the influence of fat-free-mass (FFM) on mREE was evaluated using multiple linear regression.

**Results:**

We included 292 patients (146 [50%] with body composition measurements), of which 218 (75%) patients had multifactorial obesity and 74 (25%) an underlying medical cause: non-syndromic and syndromic genetic (n= 29 and 28, respectively), hypothalamic (n= 10), and medication-induced (n= 7) obesity. Mean age was 10.8 ± 4.3 years, 59% were female, mean BMI SDS was 3.8 ± 1.1, indicating severe obesity. Mean REE% was higher in children with non-syndromic genetic obesity (107.4% ± 12.7) and lower in children with hypothalamic obesity (87.6% ± 14.2) compared to multifactorial obesity (100.5% ± 12.6, both p<0.01). In 9 children with pseudohypoparathyroidism type 1a, mean REE% was similar (100.4 ± 5.1). Across all patients, mREE was decreased in 60 (21%) patients and elevated in 69 (24%) patients. After adjustment for FFM, mREE did not differ between patients within each of the subgroups of underlying medical causes compared to multifactorial obesity (all p>0.05).

**Conclusions:**

In this cohort of children with severe obesity due to various etiologies, large inter-individual differences in mREE were found. Consistent with previous studies, almost half of patients had decreased or elevated mREE. This knowledge is important for patient-tailored treatment, e.g. personalized dietary and physical activity interventions and consideration of pharmacotherapy affecting central energy expenditure regulation in children with decreased mREE.

## Introduction

Pediatric obesity has become one of the major global health challenges of our time (1). Obesity is a complex, multifactorial disease that is caused by a chronic imbalance between energy intake and expenditure (2). Early-onset severe obesity (defined (3) as an age- and sex-specific BMI corresponding to an adult BMI of ≥35 kg/m^2^ with onset before age 5 years) can be caused by underlying medical conditions (4). These conditions can arise from disruptions in the hypothalamic regulation of hunger, satiety and energy expenditure, e.g. the leptin-melanocortin pathway (5). The current international guideline for pediatric obesity by the Endocrine Society (ES) distinguishes the following potential underlying medical causes of obesity: endocrine disorders; non-syndromic and syndromic genetic obesity disorders; weight-inducing medication; and hypothalamic dysfunction caused by hypothalamic damage, for example due to a tumor, surgery or irradiation (6).

Knowledge of an individual’s daily caloric needs is an essential part of a patient-tailored obesity management approach which supports long-term weight loss and weight maintenance (7). Total energy expenditure (TEE) is the amount of energy that individuals use on a daily basis (8). The most important contributor to TEE is resting energy expenditure (REE), which is defined as the energy required to maintain physiological homeostasis while fasting and accounts for 50-70% of TEE (7–9). The other main contributors to TEE are physical activity, linear growth and thermic effects of food intake and digestion (10). TEE can be measured using doubly-labeled water, but as this is expensive and difficult, it is often not feasible in clinical practice (8). Instead, in daily clinical practice, TEE is calculated by assessing REE, after which TEE is calculated by multiplying REE with estimated physical activity level based on the child’s age, sex, and physical activities by history taking (11–13). In practice, REE is often calculated using validated prediction equations based on age, sex, and anthropometrics. However, studies have shown that these prediction equations lack accuracy, which can lead to overestimation or underestimation of daily caloric needs and could hinder adequate obesity treatment (7). Therefore, indirect calorimetry is the gold standard for measuring REE in clinical practice which then can be used to calculate TEE and to eventually provide a patient-tailored dietary advice (14–16). Indirect calorimetry measures oxygen consumption and carbon dioxide production using a calibrated and validated metabolic cart under strictly controlled conditions. Subsequently, energy expenditure is calculated based on the individual’s oxygen consumption and carbon dioxide production using standard formulas (17).

In individuals with and without obesity, fat-free mass (FFM) is the most important contributor to REE, accounting for approximately 60-80% of the variation in REE (8, 9). In line with this, absolute REE (in kcal/day) is increased in children and adolescents with obesity compared to without obesity, but REE adjusted for FFM does not differ (8, 18, 19). For children with underlying medical causes of obesity, REE characteristics are less well described. A decreased REE is thought to be the major contributor to obesity in children with pseudohypoparathyroidism type 1a (PHP1a), a syndromic genetic obesity disorder (20, 21). Studies in children with Prader-Willi syndrome (PWS), one of the most common forms of syndromic genetic obesity, show that their reduced REE can be explained by the reduced FFM associated with the syndrome (22, 23). Furthermore, in children with hypothalamic obesity due to hypothalamic lesions or damage after surgery or radiotherapy, REE is lower compared to children with multifactorial obesity even after adjustment for FFM (24–26). However, differences in REE and body composition characteristics of children and adolescents with early-onset severe obesity with different underlying medical conditions affecting hypothalamic weight regulation have not yet been described within one cohort. As these conditions all affect the hypothalamic pathways that regulate energy expenditure, knowledge of their REE characteristics could improve patient-tailored treatment in these patients.

The aim of this study was to investigate REE in relation to body composition in children and adolescents with early-onset severe obesity with or without the following underlying medical causes: non-syndromic and syndromic genetic obesity disorders, obesity caused by hypothalamic dysfunction after hypothalamic damage, and medication-related obesity.

## Materials and Methods

**Figure 1:**
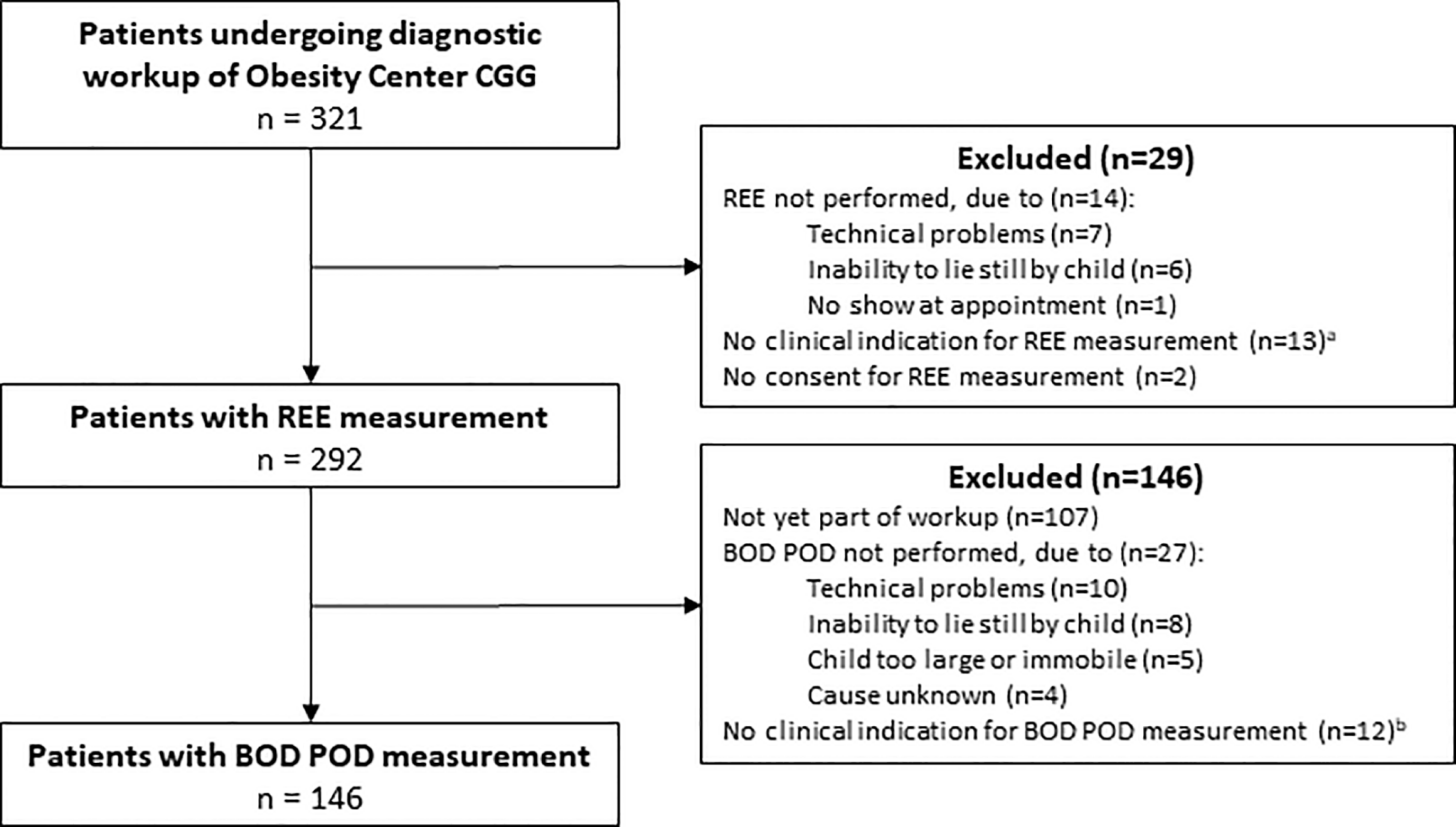
Study flow diagram. CGG, ‘Centrum Gezond Gewicht’ (in English: ‘Center for Healthy Weight’); REE, resting energy expenditure. ^a^Examples of no clinical indication for REE measurement were: REE already performed elsewhere or patient being too young for reliable measurement; ^b^Examples of no clinical indication for BOD POD measurement were: body composition already measured using dual energy X-ray absorptiometry or patient not suitable for reliable measurement e.g. due to severe intellectual disability.

For this prospective observational study, we used data of children (up to 19 years) visiting the outpatient clinic of Obesity Center CGG, a Dutch referral center for obesity, at the academic center Erasmus MC-Sophia Children’s Hospital (Rotterdam, The Netherlands) between April 2014 and April 2021. Pediatric patients were referred to Obesity Center CGG for diagnostic evaluation of their early-onset severe obesity due to suspicion of underlying medical causes and/or personalized therapeutic advices (4). All consecutive patients in whom REE was measured using indirect calorimetry as part of the standardized diagnostic workup of Obesity Center CGG were included in this study (4). Exclusion criteria were inability or refusal to give informed consent or not completing the REE measurement (**Figure 1**). This study was approved by the medical ethics committee of the Erasmus MC (MEC-2012-257). All parents/caretakers of children ≤16 years gave written informed consent. Additionally, children aged ≥12 years also gave written informed consent; children aged ≤12 years also gave oral assent.

### Assessment of Underlying Medical Causes of Obesity

The standardized diagnostic approach of Obesity Center CGG consists of two visits: (1) an initial visit during which patients are screened by a pediatric endocrinologist following Dutch and international guidelines for pediatric obesity. This includes extensive medical history taking, physical examination, and detailed growth charts assessment (6, 27); (2) a subsequent visit where patients return after an overnight fast for indirect calorimetry, body composition assessment and blood sampling including biochemical and hormonal assessment and extensive genetic testing (obesity gene panel, microarray analysis) (4). Height and weight were measured and BMI was calculated rounded to the nearest decimal by trained personnel and converted to age- and sex-specific standard deviation scores (SDS) using Dutch growth charts (28). The standardized diagnostic approach has previously been described in further detail (4). After the diagnostic approach was completed, patients were classified in the following groups based on the presence or absence of underlying medical causes of obesity:

- Endocrine disorders: endogenous Cushing’s syndrome or clinical hypothyroidism

- Non-syndromic and syndromic genetic obesity disorders: diagnosed when genotyping revealed known (likely) pathogenic variants [as defined by the American College of Medical Genetics and Genomic guideline (29)] in obesity-associated genes which matched the clinical phenotype (4). Classification of genetic obesity disorders was based on the Endocrine Society’s guideline for pediatric obesity (6)

- Medication-related obesity: start or intensification of known weight-inducing medication coinciding with development or progression of obesity in the patient’s growth charts in the absence of other plausible explanations for the sudden weight gain (4)

- Hypothalamic obesity: central nervous system (CNS) injury affecting the hypothalamic region that regulates satiety and energy expenditure due to congenital anatomical defects, tumor (e.g. craniopharyngioma), surgery, irradiation, meningitis or ischemic damage, coinciding with development or progression of obesity in the patient’s growth charts in the absence of other plausible explanations for the sudden weight gain (4)

- Multifactorial obesity: obesity due to a combination of lifestyle, environmental and genetic background; abovementioned underlying medical causes were excluded in the extensive diagnostic workup.

### REE Measurement

REE measurements were performed using indirect calorimetry with a metabolic cart (Quark RMR, COSMED, Italy). Patients had fasted overnight (at least 8 hours) and did not perform physical activity prior to the measurement. The Quark RMR was calibrated according to the manufacturer’s recommendations. The first 5 minutes of the measurement were excluded from the results to allow acclimation. The aim was to obtain measured REE (mREE) after 15 minutes of measurement in steady state (VCO2 coefficient of variation [CV%] and VO2 CV% both <10) (30). Measured REE was calculated based on VO2 and VCO2 using the Weir equation (17). If possible, considering the child’s age and ability to lie still for at least 20 minutes, the measurement was performed without distraction with a book or screen. For children aged <18 years, the Schofield equations were used to calculate predicted REE (pREE), as a recent systematic review concluded that these provided the most accurate (smallest difference between mREE and pREE) REE predictions in children and adolescents with obesity (7). The original equations by Schofield were used with application of a conversion factor of 239.006 to transform megajoules to kilocalories (31). For patients aged ≥18 years at REE measurement, the 1984 Harris & Benedict equations were used as these were shown to be the most accurate in adults with obesity (32). As a sensitivity analysis, we also calculated pREE based on the equations by Molnár (33), as a recent large external validation study found that these equations had the best precision (highest proportion of children with pREE within 90-110% of mREE) in children with obesity (16). Since the Schofield and Molnár equations are based on body weight, we also performed a sensitivity analyses using body composition-based prediction equations specifically designed for children with severe obesity (Lazzer equations) (34).

### Body Composition Measurement

From March 2018 onwards, the standardized diagnostic workup of our obesity center also included body composition measurement using air displacement plethysmography (BOD POD, COSMED, Italy). The BOD POD was warmed up and calibrated according to the manufacturer’s instructions. Thoracic volume was predicted by the BOD POD software (35, 36). Patients were instructed to wear swimwear or tight underwear and a swim cap during the measurement. Two-compartment body composition (fat-free mass; FFM and fat mass; FM) was determined from body volume using density model Lohman according to the manufacturer’s recommendation for children (37).

### Statistical Analyses

Statistical analyses were performed using SPSS version 25.0 (Armonk, NY: IBM Corp.) and GraphPad Prism version 8 (GraphPad Software, Inc.). Data are presented as median (interquartile range; IQR), or mean (standard deviation; SD), as appropriate. The bias between mREE and pREE (mREE – pREE) in kcal/day and ratio between mREE and pREE (mREE/pREE * 100%; REE%) were calculated, with normal mREE defined as REE% between 90-110% of predicted, decreased mREE defined as REE% ≤90% and elevated mREE defined as REE% ≥110% (7). Bivariate correlations between mREE and FFM, and REE% and age and BMI SDS were assessed across all patients and in each subgroup of underlying medical causes separately using Pearson’s *r* (if sample size ≥25 patients) or Kendall’s tau (if sample size between 10-25 patients). The effect of sex and ethnicity on mREE and REE% were assessed using multiple linear regression analyses. For mREE, pairwise comparisons between each of the underlying medical causes versus multifactorial obesity were performed in separate regression analyses (e.g. non-syndromic genetic vs multifactorial, syndromic genetic vs multifactorial, etc.) with adjustments for FFM, FM, and sex. In each regression analysis, the grouping variable was defined as multifactorial obesity =0, underlying cause =1. The difference in slope was tested by including the interaction term underlying cause x FFM. For the regression models with hypothalamic obesity and medication-induced obesity, only the main effect and interaction effect of the underlying cause were entered in the regression models to prevent overfitting. Furthermore, pairwise comparisons were made between REE and body composition characteristics of children with each of the underlying medical causes versus children with multifactorial obesity using unpaired t-tests, Mann-Whitney tests, or chi-squared tests, as appropriate. A Bland-Altman analysis was performed to investigate agreement between mREE and pREE. To investigate proportionality of bias, linear regression analyses with and without adjustment for the presence of underlying medical causes were performed using the bias between mREE and pREE as independent variable and the mean of mREE and pREE as dependent variable. These analyses were performed using the absolute difference between mREE and pREE (mREE – pREE) as well as the relative difference ((mREE – pREE)/(mean of mREE and pREE) * 100%). Finally, since movement and/or agitation during the REE measurement can cause falsely elevated mREE values, we performed sensitivity analyses using only REE measurements in which an optimal steady state was achieved (30). For these sensitivity analyses, only REE measurements with a fractional concentration of CO2 (FeCO2) >0.5, a measurement duration of at least 5 minutes, and a CV% of <10% for both VO2 and VCO2 were included (30). For all statistical analyses, two-sided P-values <0.05 were considered statistically significant.

## Results

In total, n=292 patients were included (**Figure 1**), of which 218 (75%) had multifactorial obesity and 74 (25%) had an underlying medical cause (**Table 1**). This included non-syndromic genetic obesity in 29 (10%) patients, syndromic genetic obesity in 28 (10%) patients, hypothalamic obesity in 10 (3%) patients, and medication-induced obesity in 7 patients (2%; **Table 1**). The mean age of included patients was 10.8 ± 4.3 years (**Table 2**). A majority of 172 (59%) patients were female. The mean BMI SDS across all participants was 3.76 ± 1.07, indicating severe obesity. The BOD POD measurement was performed in 146 (50%) patients. Children for whom a BOD POD measurement was available were slightly older than children without a BOD POD measurement, but this group did not differ with regard to other baseline characteristics (**Supplementary Table S1**).

**Table 1:** Diagnosed underlying medical causes of obesity in the study population.

Diagnosis category	Number of patients	Details
Non-syndromic genetic obesity	29 (10%)	18 (60%) Heterozygous melanocortin 4 receptor (*MC4R)* deficiency 6 (20%) Biallelic leptin receptor (*LEPR)* deficiency 3 (10%) Heterozygous proopiomelanocortin (*POMC)* deficiency 1 (3%) Heterozygous proprotein convertase subtilisin/kexin type 1 (*PCSK1)* deficiency 1 (3%) Biallelic *MC4R* deficiency
Syndromic genetic obesity	28 (10%)	9 (32%) 6 (22%) 16p11.2 deletion syndrome 5 (19%) Bardet-Biedl syndrome 3 (11%) Temple syndrome 2 (7%) 1 (4%) Alström syndrome 1 (4%) Cohen syndrome 1 (3%) 6q16.3 deletion including *SIM1*
Hypothalamic obesity	10 (3%)	4 (40%) after surgery and/or radiotherapy for intracranial tumors 2 (20%) in presence of myelomeningocele 1 (10%) after ischemic stroke 1 (10%) after neonatal meningitis 1 (10%) in presence of Chiari I malformation, ectopic neurohypophysis and pituitary hormone deficiencies 1 (10%) in presence of panhypopituitarism, hyperphagia and central precocious puberty, highly suspicious for hypothalamic dysfunction
Medication-induced obesity	7 (2%)	5 (71%) induced by corticosteroids 1 (14%) induced by anti-epileptics 1 (14%) induced by anti-psychotics
Endocrine disorders	0 (0%)	–
Multifactorial obesity	218 (75%)	No singular underlying medical cause of obesity

**Table 2:** Baseline characteristics of the study population.

	All patients (n=292)	Non-syndromic genetic obesity (n=29)	Syndromic genetic obesity (n=28)	Hypothalamic obesity (n=10)	Medication-induced obesity (n=7)	Multifactorial obesity (n=218)
Age, years	10.8 (4.3)	10.5 (4.4)	10.8 (4.6)	14.0 (2.6)*	11.2 (3.5)	10.7 (4.2)
Sex, female, n (%)	172 (59)	19 (66)	19 (68)	7 (70)	3 (43)	124 (57)
Ethnicity, Dutch, n (%)	202 (69)	21 (72)	21 (75)	7 (70)	1 (14)*	152 (70)
Height, cm	147.4 (23.4)	150.3 (30.6)	142.2 (19.6)	156.2 (11.8)	150.6 (18.3)	147.2 (23.3)
Height SDS	0.33 (1.39)	1.01 (1.12)*	-0.32 (1.56)*	-0.93 (0.87)**	0.20 (0.99)	0.39 (1.37)
Weight, kg	72.6 (33.2)	81.3 (39.2)	59.8 (26.8)	80.7 (17.4)	72.4 (21.6)	72.7 (33.7)
Weight SDS	3.70 (1.53)	4.32 (1.22)*	2.81 (1.63)**	2.96 (1.11)*	3.65 (0.39)	3.77 (1.54)
BMI, kg/m^2^	31.2 (7.4)	33.1 (6.8)	28.0 (7.1)*	32.8 (4.4)	31.2 (3.5)	31.3 (7.6)
BMI SDS	3.76 (1.07)	4.12 (1.07)	3.23 (1.30)*	3.42 (0.57)	3.89 (0.50)	3.79 (1.05)

BMI, body mass index; SDS, standard deviation score. Data presented as mean (SD), unless otherwise stated. *P < 0.05 **P < 0.01 vs multifactorial obesity.

### REE and Body Composition Characteristics

The REE and body composition characteristics of the study population are presented in **Table 3**. Mean mREE was lower in children with syndromic genetic obesity compared to children with multifactorial obesity (1479 ± 360 vs 1719 ± 490 kcal/day, p<0.05). The mean percentage of FFM across all patients was 55.2% ± 8.1 and did not differ between patients with underlying medical causes of obesity and patients with multifactorial obesity (p-values >0.05; **Table 3**). When expressed in absolute values and adjusted for sex, age, and BMI SDS, FFM was higher compared to multifactorial obesity in children with non-syndromic genetic obesity (adjusted regression coefficient +6.8kg FFM, SE 1.91, p<0.001), but lower in children with syndromic genetic obesity (adjusted regression coefficient -5.3kg FFM, SE 2.23, p=0.02), hypothalamic obesity (adjusted regression coefficient -11.7kg FFM, SE 3.44, p<0.001) and similar in medication-induced obesity (adjusted regression coefficient +2.4kg FFM, SE 5.6, p=0.67).

**Table 3:** REE and body composition characteristics of the study population.

	All patients (n=292)	Non-syndromic genetic obesity (n=29)	Syndromic genetic obesity (n=28)	Hypothalamic obesity (n=10)	Medication-induced obesity (n=7)	Multifactorial obesity (n=218)
mREE, kcal/day	1705 (491)	1884 (612)	1479 (360)*	1535 (236)	1710 (342)	1719 (490)
pREE, kcal/day	1718 (522)	1777 (614)	1511 (360)*	1780 (324)	1821 (387)	1730 (534)
Mean bias (mREE – pREE), kcal/day	-12 (240)	107 (231)*	-32 (150)	-245 (270)**	-111 (365)	-12 (236)
REE%	100.4 (12.8)	107.4 (12.7)**	99.5 (13.3)	87.6 (14.2)**	95.5 (17.1)	100.5 (12.6)
Lowered mREE, n (%)	60 (21)	3 (10)	6 (21)	6 (60)**	2 (29)	41 (19)
Elevated mREE, n (%)	69 (24)	12 (41)	0 (0)**	1 (10)	2 (29)	54 (25)
FFM, %BW	55.2 (8.1)^a^	56.2 (5.9)^a^	57.1 (8.6)^a^	48.2 (11.2)^a^	56.2 (10.3)^a^	55.1 (8.2)*^a^*

mREE, measured resting energy expenditure; pREE, predicted resting energy expenditure (based on Schofield equations); REE%, ratio mREE/pREE; FFM, fat-free mass; %BW, percentage of body weight; kcal, kilocalories. Data presented as mean (SD), unless otherwise stated. ^a^ Available for n=146 patients with available BOD POD measurement (18 non-syndromic, 13 syndromic, 5 hypothalamic, 2 medication-induced, and 108 multifactorial obesities) *P < 0.05 **P < 0.01 vs multifactorial obesity.

Across all patients, mREE was positively associated to FFM (*r* = 0.85, p<0.001). REE% was not associated with age (*r =* -0.06, p=0.26) nor with BMI SDS (*r* = -0.09, p=0.14; **Supplementary Figure S1**). Subgroup analyses stratified on underlying medical causes revealed no major differences in the presence or absence and magnitude of these associations (**Supplementary Table S2**).

In linear regression analyses adjusting for FFM and FM, mREE was associated with sex (females vs males -148 kcal/day, SE 36.3, p<0.001), but not ethnicity (non-Dutch vs Dutch -52.3 kcal/day, SE 40.3, p=0.20). After adjustment for body composition, mREE did not differ between patients with each of the underlying medical causes compared to patients with multifactorial obesity (p-values of main effects and interaction effects all >0.05, **Table 4**; **Supplementary Figure S2**).

**Table 4:** Results of multiple regression analyses on differences in mREE (kcal/day) between patients with each of the underlying medical causes versus multifactorial obesity.

Non-syndromic genetic vs multifactorial (n=126, R^2^ = 0.83)				
	Coefficient	SE	95% CI	p-value
FFM (kg)	13.85	2.13	9.64; 18.06	<0.001
FM (kg)	11.45	1.78	7.93; 14.97	<0.001
Sex, female	-180.90	37.79	-255.71; -106.07	<0.001
Non-syndromic genetic	17.14	158.95	-297.58; 331.85	0.91
Non-syndromic genetic x FFM	3.03	3.41	-3.73; 9.78	0.38
**Syndromic genetic vs multifactorial (n=121, R^2^ = 0.82)**				
	**Coefficient**	**SE**	**95% CI**	**p-value**
FFM (kg)	14.17	2.15	9.90; 18.43	<0.001
FM (kg)	11.25	1.80	7.69; 14.81	<0.001
Sex, female	-150.81	38.62	-227.32; -74.30	<0.001
Syndromic genetic	-54.38	179.55	-410.04; 301.28	0.76
Syndromic genetic x FFM	-1.47	4.87	-11.12; 8.19	0.76
**Hypothalamic vs multifactorial (n=113, R^2^ = 0.72)**				
	**Coefficient**	**SE**	**95% CI**	**p-value**
FFM (kg)	25.63	1.58	22.49; 28.76	<0.001
Hypothalamic	-5.37	438.84	-875.15; 864.40	0.99
Hypothalamic x FFM	-5.16	12.11	-29.17; 18.84	0.67
**Medication-induced vs multifactorial (n=110, R^2^ = 0.71)**				
	**Coefficient**	**SE**	**95% CI**	**p-value**
FFM (kg)	25.63	1.60	22.46; 28.80	<0.001
Medication-induced	-145.81	856.88	-1844.66; 1553.05	0.87
Medication-induced x FFM	3.98	17.67	-31.06; 39.02	0.82

mREE, measured resting energy expenditure; kcal, kilocalories; CI, confidence interval; FFM, fat-free mass; FM, fat mass. Data presented as unstandardized regression coefficients (absolute difference in kcal/day adjusted for the other variables in the model). For the regression models with hypothalamic obesity and medication-induced obesity, only the main effect and interaction effect of the underlying cause were entered in the model to prevent overfitting.

### Measured REE vs Predicted REE

**Figure 2:**
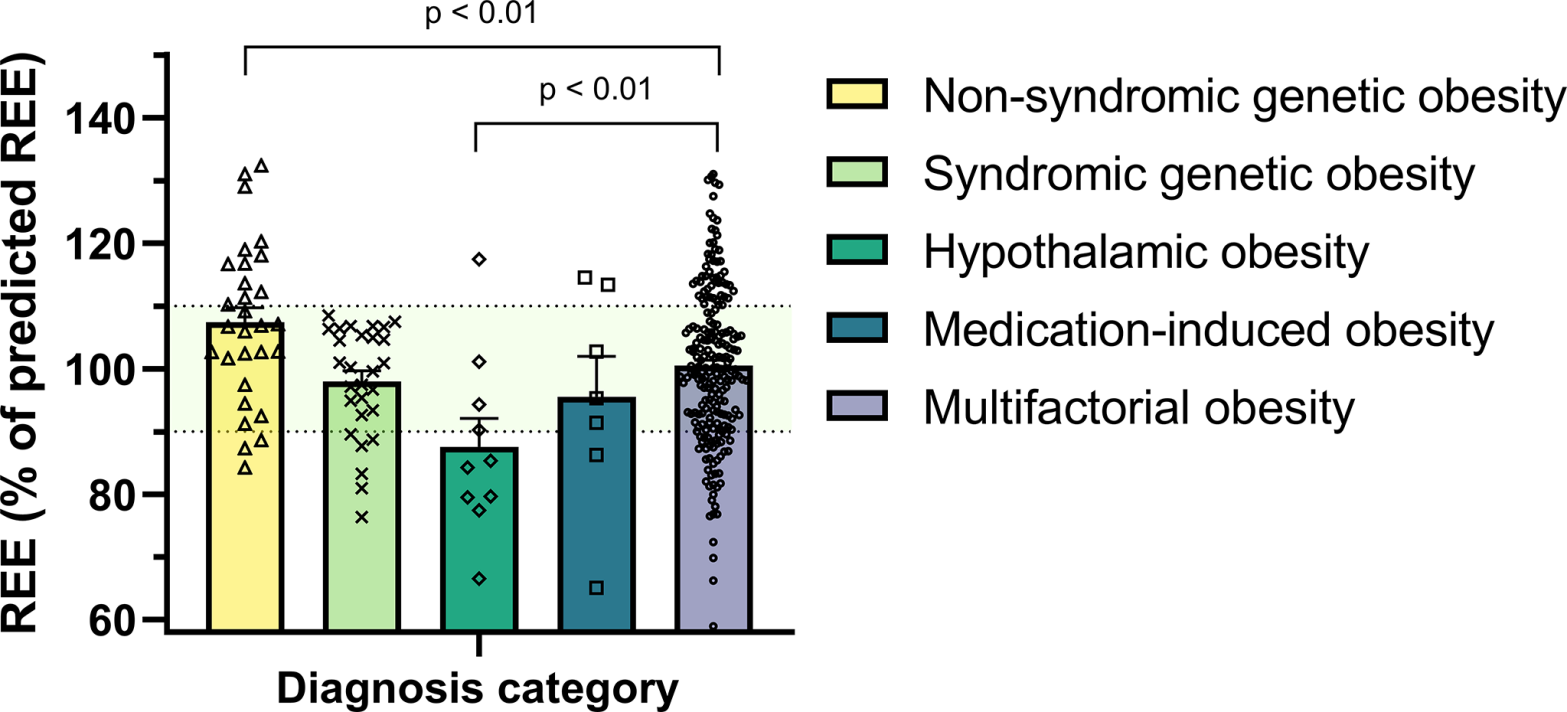
Measured REE expressed as percentage of predicted REE (by Schofield equations) across the study population. Patients with non-syndromic genetic obesity had higher REE% compared to children with multifactorial obesity whereas children with hypothalamic obesity had lower REE% (both p-values <0.01). The dots represent the individual patients. The bars represent the mean + standard error of the mean. The light green shaded area indicates a REE% between 90 and 110%. REE, resting energy expenditure.

The mean bias (absolute difference between mREE and pREE) across all patients was -12.0 ± 240 kcal/day, corresponding to a mean REE% of 100.4% ± 12.8 (**Table 3**). In linear regression analyses, REE% was associated with sex (females vs males +9.4%, SE 1.6, p<0.001) and ethnicity (non-Dutch vs Dutch -5.2%, SE 1.8, p=0.004). This indicates that the Schofield equations tend to underpredict REE in girls compared to boys and overpredict in children with non-Dutch ethnicity compared to Dutch ethnicity. Children with non-syndromic genetic obesity had a positive mean bias and higher REE% compared to children with multifactorial obesity (mean bias +107 ± 231 kcal/day vs -12 ± 236 kcal/day; mean REE% 107.4% ± 12.7 vs 100.5% ± 12.6, both p<0.01, **Table 3** and **Figure 2**). On the other hand, children with obesity due to hypothalamic dysfunction showed a negative mean bias and lower REE% compared to children with multifactorial obesity (mean bias -245 ± 270 kcal/day; mean REE% 87.6% ± 14.2, both p<0.01, **Figure 2**). Similarly, children with medication-induced obesity showed negative mean bias and lower REE% compared to children with multifactorial obesity, although the differences did not reach statistical significance (**Table 3**). These results remained similar after stratification on sex and ethnicity (**Supplementary Figures S3**, **S4**).

### Decreased mREE

Sixty (21%) patients had a decreased mREE (mREE ≤90% of pREE), of which 3 patients with non-syndromic genetic obesity (a pathogenic heterozygous *MC4R* variant in 2 patients and a heterozygous *PCSK1* variant in one patient; **Table 5**), 6 patients with syndromic genetic obesity (two 16p11.2 deletion syndrome, 1 Bardet-Biedl syndrome, 1 Cohen syndrome, 1 PHP1b, 1 Temple syndrome; **Table 5**), 6 patients with obesity caused by hypothalamic dysfunction, 2 patients with medication-induced obesity, and 43 patients with multifactorial obesity. The proportion of children with hypothalamic obesity with decreased mREE was higher than in children with multifactorial obesity (6/10, 60% vs 41/216, 19%; p<0.01). The mean bias between mREE and pREE in the children with decreased mREE was -341 ± 198 kcal/day. This indicates that the Schofield equations would overestimate REE in these children by on average 341 kcal/day compared to mREE.

**Table 5:** Overview of clinical and REE characteristics of patients with genetic obesity disorders who had a decreased REE.

Patient	Gene/CNV	Reference transcript	Genetic alteration	Age in years	Sex	BMI SDS	mREE (kcal/day)	REE%^a^	FFM (kg)	FM (kg)
Non-syndromic genetic obesity										
1	*MC4R*	NM_005912.2	Heterozygous c.913C>T p.(Arg305Trp)	7.2	Male	5.15	1371	84.2	–	–
2	*MC4R*	NM_005912.2	Heterozygous c.105C>A p.(Tyr35*)	9.4	Female	3.90	1593	87.3	37.1	33.9
3	*PCSK1*	NM_000439.4	Heterozygous c.541T>C p.(Tyr181His)^b^	12.3	Female	3.55	1409	88.6	–	–
Syndromic genetic obesity										
4	*Epigenetic error chr20* (PHP1b)	n/a	Imprinting defect on paternal allele of chromosome 20 leading to sporadic pseudohypoparathyroidism type 1b	3.2	Male	3.59	842	88.7	13.6	7.1
5	Del16p11.2	n/a	Deletion chromosome 16p11.2 (hg19: 28,843,890_29,044,745)x1	8.10	Female	4.26	1398	89.6	32.4	24.8
6	Del16p11.2	n/a	Deletion chromosome 16p11.2 (hg19: 29,627,349_30,199,713)x1	18.4	Male	3.92	1720	80.9	56.0	44.7
7	*BBS10* (Bardet-Biedl syndrome)	NM_005912	Homozygous c.271dupT p.(C91Leufs*5), leading to Bardet-Biedl syndrome	15.0	Male	3.90	2001	83.3	46.3	54.7
8	*Epigenetic error chr14* (Temple syndrome)	n/a	Imprinting defect on chromosome 14 leading to Temple syndrome	8.2	Female	3.53	1269	87.7	–	–
9	*VPS13B* (Cohen syndrome)	NM_017890.4	Compound heterozygous c.2911C>T p.(Arg971*), c.8697-2A>G p.?, leading to Cohen syndrome	8.6	Male	2.22	968	76.4	–	–

^a^ predicted REE based on Schofield equations (for children <18 years) or 1984 Harris & Benedict equations (adolescents ≥18 years) ^b^ risk factor for early-onset obesity; n/a, not applicable; -, not available (no BOD POD measurement performed). CNV, copy number variation; SDS, standard deviation score; REE, resting energy expenditure; kcal, kilocalories; REE%, ratio measured REE/predicted REE; FM, fat mass; FFM, fat-free-mass; PHP1b, pseudohypothyroidism type 1b.

### Elevated mREE

In 69 (24%) patients an elevated mREE (mREE ≥110% of predicted) was found, most of which had multifactorial obesity (n=54) or non-syndromic genetic obesity (n=12); only one patient had hypothalamic obesity and two patients had medication-induced obesity. The highest proportion of elevated mREE was found in children with non-syndromic genetic obesity (12/29 patients, 41%), which was higher than the proportion of children with multifactorial obesity with elevated mREE (54/218, 25%, p<0.05)

### REE Characteristics in Genetic Obesity Syndromes

When zooming in on the 9 children with PHP1a, a genetic obesity syndrome which has previously been associated with decreased REE, these children showed a mean REE% of 100.4 ± 5.1 and similar mREE adjusted for FFM (available for 6 patients) compared to children with multifactorial obesity (coefficient -37.5 kcal/day, SE 119.0, p=0.75). Furthermore, none of the children with PHP1a had a decreased mREE (p=0.21 compared to children with multifactorial obesity). In contrast, a decreased REE was found in 2 out of 6 (33%) children with 16p11.2 deletion syndrome and 1 out of 3 (33%) children with Temple syndrome, two genetic obesity syndromes of which REE characteristics have not yet been described. The 6 children with 16p11.2 deletion syndrome had a mean REE% of 99.5 ± 11.4 and similar mREE adjusted for FFM (available for 3 patients) compared to children with multifactorial obesity (coefficient -212.3 kcal/day, SE 152.3, p=0.17). In the 3 children with Temple syndrome, mean REE% was 99.7 ± 10.4 (p>0.05 compared to children with multifactorial obesity); these 3 children did not have a BOD POD measurement available. In the five children with Bardet-Biedl syndrome, mean REE% was 96.5 ± 8.6 and mREE adjusted for FFM (available for 2 patients) was similar compared to children with multifactorial obesity (coefficient 28.4 kcal/day, SE 151.4, p=0.85).

### Bland-Altman Analyses

**Figure 3:**
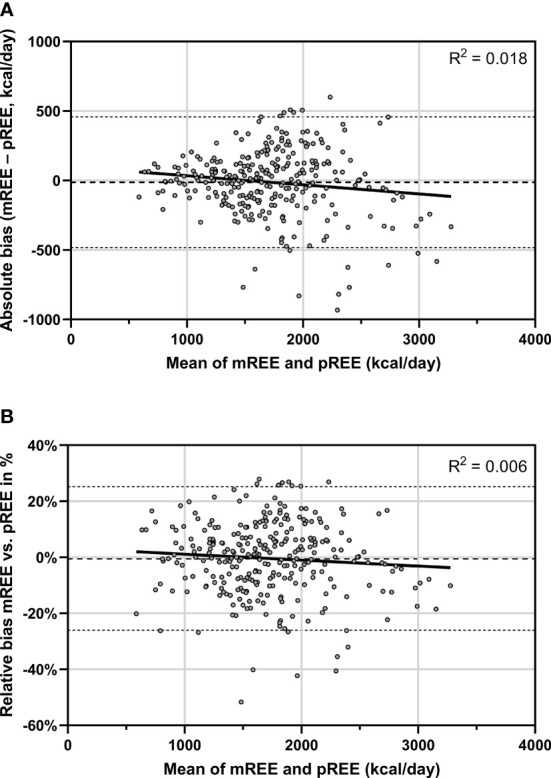
Bland-Altman plot for the agreement between mREE and pREE (by Schofield equations). The dots represent the individual patients. The middle dashed line represents the mean absolute **(A)** or relative bias **(B)** across the study population. The upper and lower dashed lines represent the upper and lower limits of agreement (mean bias ± 1.96 SD) of mREE and pREE. The solid line represents the linear regression fit line. mREE, measured resting energy expenditure; pREE, predicted resting energy expenditure (using the Schofield equations).

The Bland-Altman plot of mREE vs pREE is presented in **Figure 3**. When expressing the bias in absolute numbers (mREE – pREE in kcal/day), the limits of agreement were -482 kcal to +457 kcal/day. A statistically significant negative relation was found between the mean of mREE and pREE and the absolute bias between mREE and pREE (unstandardized regression coefficient -0.066 kcal/day, SE=0.028, p=0.02, **Figure 3A**). This indicates that with increasing values for the mean of mREE and pREE, the absolute negative bias between mREE and pREE becomes larger. This negative relationship remained similar after adjustment for presence of underlying causes (unstandardized regression coefficient -0.076, SE=0.028, p=0.007). However, when expressing the bias in relative difference, this negative relationship was no longer present (unstandardized regression coefficient -0.0021%, SE 0.0016, p=0.17, **Figure 3B**), also after adjustment for presence of underlying causes (unstandardized regression coefficient -0.0028%, SE 0.0015, p=0.07). The mean relative bias was -0.42% with limits of agreement of –26% to +25%.

### Sensitivity Analyses

Sensitivity analyses using only REE measurements in which an optimal steady state was achieved (n=172 measurements) showed similar numerical results with regard to REE and BOD POD characteristics. Most differences between the subgroups were no longer statistically significant, probably due to the smaller sample sizes (**Supplementary Table S3**). When restricting these analysis to patients in whom body composition was measured, again similar numerical results were found without statistically significant differences (**Supplementary Table S4**).

Sensitivity analyses using the Molnár equations to calculate pREE (pREE_Molnár_) showed similar results with regard to differences in REE characteristics between patients with underlying medical causes of obesity and patients with multifactorial obesity (**Supplementary Table S5**). Interestingly, pREE_Molnár_ underestimated mREE in almost all patient subgroups with an average mean bias ranging between +55 and +131 kcal/day across the patient subgroups, except for patients with hypothalamic obesity, who had a mean bias of -116 ± 201 kcal/day (p<0.01 vs multifactorial obesity). This resulted in a mean REE% of 105.1% ± 13.6 in the total study population and a higher proportion of patients with an elevated mREE (37% vs. 24%) and a lower proportion of patients with a decreased mREE (12% vs. 21%) when compared to the results using the Schofield equations to calculate pREE (**Supplementary Table S5**). REE%_Molnár_ was associated with sex (females vs males -5.6%, SE 1.6, p=0.001) but not with ethnicity (non-Dutch vs Dutch -0.1%, SE 1.8, p=0.95), indicating that the Molnár equations tend to overpredict REE in girls compared to boys. Bland-Altman analyses using the Molnár equations showed a statistically significant positive relation between the mean of mREE and pREE_Molnár_ and the absolute bias between mREE and pREE_Molnár_ (unstandardized regression coefficient 0.14 kcal/day, SE 0.026, p<0.001; **Supplementary Figure S5a**). This indicates that with increasing values for the mean of mREE and pREE, the absolute positive bias between mREE and pREE becomes larger. Adjustment for underlying causes showed similar results (p<0.001). The relative bias also showed a small but statistically significant positive association with the mean of mREE and pREE_Molnár_ (unstandardized regression coefficient 0.007%, SE 0.0017, p<0.001; **Supplementary Figure S5b**), which remained similar after adjustment for underlying causes (p<0.001).

Sensitivity analyses using the body-composition based Lazzer equations to calculate pREE (pREE_Lazzer_) also showed similar results (**Supplementary Table S6**). On group level, the mean absolute bias between mREE and pREE_Lazzer_ was -21 kcal, resulting in an average REE% of 98.5% ± 12.1. Moreover, similar results were found with regard to differences in REE characteristics between patients with underlying medical causes of obesity and patients with multifactorial obesity: patients with non-syndromic genetic obesity had higher REE% (105.0% ± 9.4) than children with multifactorial obesity (98.7% ± 12.0) whereas children with hypothalamic obesity had lower REE% (86.6% ± 3.7, both p<0.05). REE%_Lazzer_ was associated with sex (females vs males +5.3%, SE 2.0, p=0.008) but not with ethnicity (non-Dutch vs Dutch -3.1%, SE 2.3, p=0.18), indicating that the Lazzer equations tend to underpredict REE in girls compared to boys. Bland-Altman analyses using the Lazzer equations showed a statistically significant positive relation between the mean of mREE and pREE_Lazzer_ and the absolute bias between mREE and pREE_Lazzer_ (unstandardized regression coefficient 0.15 kcal/day, SE 0.038, p<0.001; **Supplementary Figure S6a**). This indicates that with increasing values for the mean of mREE and pREE, the absolute positive bias between mREE and pREE becomes larger. Adjustment for underlying causes showed similar results (p<0.001). The relative bias also showed a small but statistically significant positive association with the mean of mREE and pREE_Lazzer_ (unstandardized regression coefficient 0.011%, SE 0.0022, p<0.001; **Supplementary Figure S6b**), which remained similar after adjustment for underlying causes (p<0.001).

## Discussion

This study presents the REE and body composition characteristics of a cohort of children with early-onset severe obesity with and without a diagnosis of underlying medical disorders that affect the hypothalamic regulation of satiety and energy expenditure. On a group level, measured REE seems to match predicted REE quite accurately, with a mean bias across the study population of -12 kcal/day and a mean measured REE of 100.5% of predicted values. However, our main finding is that large inter-individual and between-disorder differences between measured and predicted REE were found across all subgroups of patients. Almost half of the patients showed measured REE that was ≥10% decreased or elevated compared to predicted REE. In the 21% of patients with a decreased measured REE, the mean difference between measured and predicted REE was -341 kcal/day. The highest proportion of decreased REE was found in children with hypothalamic obesity, who on average had a measured REE of 87.6% of predicted values. The strong association between measured REE and FFM (available in 50% of patients) was similar across all patient groups with and without underlying causes. Moreover, no differences were found in measured REE adjusted for FFM between children with underlying medical causes of obesity compared to children with multifactorial obesity. Thus, our study underlines the importance of measuring REE and relating the values to body composition in all children with early-onset severe obesity with or without a diagnosis of underlying medical causes that affect hypothalamic weight regulation.

In the past decades, several studies that concomitantly measured both TEE as well as REE in children with obesity concluded that reduced REE on its own is not the major cause of common obesity (8, 38, 39). Although some studies have investigated REE in specific patient subgroups with underlying medical causes of obesity, our study is to our knowledge the first to investigate REE and body composition characteristics in a relatively large cohort of children with early-onset severe obesity due to various underlying medical causes that can affect the central homeostatic maintenance of energy balance. The hypothalamic leptin-melanocortin system is a key element of the regulation of hunger, satiety and energy balance (5). The main downstream effector is the melanocortin-4 receptor (MC4R), which upon stimulation by its endogenous ligand α-MSH promotes satiety and increases energy expenditure, whereas antagonism of MC4R action increases food intake and energy conservation (40). In the current report, we studied children with non-syndromic and syndromic genetic obesity disorders, hypothalamic damage and weight-inducing medication as models of hypothalamic obesity and investigated REE and body composition characteristics compared to children with multifactorial early-onset severe obesity.

### Multifactorial Obesity

In our cohort, REE% in children with multifactorial obesity on group level matched predicted values, with a mean bias of only -12 kcal/day, corresponding to a mean REE% of 100.5%. However, the large standard deviation of REE% of 12.8% indicates that the inter-individual differences in measured versus predicted REE were considerable. Furthermore, over half of our patients with multifactorial obesity had a REE% between 90-110%. These results are in line with previous general pediatric obesity cohort studies where mean REE% ranged between 90-111% and the proportion of patients with predicted REE within 10% of measured REE using the Schofield equations ranged from 21-61% (16, 41–43). Furthermore, our study confirms that the strong association between FFM and mREE is also observed in children with severe obesity (44).

### Non-Syndromic Genetic Obesity Disorders

Our results showed that measured REE is on average +107 kcal higher than predicted REE in children with non-syndromic genetic obesity disorders. This result can be explained by the fact that these patients had more severe obesity than children with multifactorial obesity, and since BMI z-score is positively associated with FFM (45), a relatively higher FFM. Indeed, patients with non-syndromic genetic obesity had +6.8 kg higher FFM than children with multifactorial obesity after adjustment for age, sex and BMI SDS, and their mREE adjusted for FFM did not differ from children with multifactorial obesity. Thus, measuring body composition in these patients is necessary to correctly interpret their REE. Although the genetic defects of these patients interfere with hypothalamic leptin-melanocortin signalling (5), and *Mc4r* knockout mice correspondingly show reduced basal oxygen consumption (40), most studies investigating REE in humans with these rare, non-syndromic genetic obesity disorders did not find evidence for decreased REE. These studies, performed in 29 patients with MC4R deficiency (46), two (47) and eight (48) patients with LEPR deficiency and one patient with PCSK1 deficiency (49) report a normal REE. In contrast, the first two children ever to be described with biallelic *POMC* variants were found to have a decreased REE ranging between -17% and -27% compared to the Schofield equations (50). Another study in eight adult Pima Indians with heterozygous pathogenic *MC4R* variants showed on average -140 kcal/day lower REE compared to non-genetic obesity controls (51). Whether this finding, which has not been replicated in other patients with *MC4R* deficiency, is related to the specific ethnic background of these patients or unidentified factors affecting REE remains to be investigated. In our study, two patients with heterozygous pathogenic *MC4R* variants and one patient with a heterozygous *PCSK1* variant that is a risk factor for early-onset obesity (4) had a decreased REE, but the proportion of patients with decreased REE did not differ between the non-syndromic genetic obesity disorders (3/29; 10%) and the multifactorial obesity group (41/218; 19%). Together, this suggests that REE can be decreased in non-syndromic genetic obesity disorders, but not more or less often than in children with early-onset severe multifactorial obesity. Therefore, it remains important to measure REE in these patients and to not rely on predicted REE only.

### Syndromic Genetic Obesity Disorders

Contrary to our expectations, we did not find major differences in REE characteristics in syndromic genetic obesity disorders compared to patients with multifactorial obesity. Various syndromic disorders in this patient group are associated with lower lean body mass and/or muscle hypotonia (23, 52–55). Yet, it seems that the Schofield equations can accurately predict REE in these patients on group level, as these patients had an average REE% of 99.5%. Moreover, we did not find differences in mREE adjusted for FFM compared to children with multifactorial obesity, which is in line with previous studies performed in children and/or adults with Prader-Willi syndrome (22, 23, 56), Alström syndrome (52), and Bardet-Biedl syndrome (53). For other syndromic obesity disorders in our study population, namely Temple syndrome, 16p11.2 deletion syndrome and Cohen syndrome, REE characteristics have not yet been described in literature. Although we found no evidence for a decreased REE% in patients with these rare syndromic obesity disorders, it should be noted that the small sizes of these subgroups in our study population warrant further studies before any conclusions regarding REE characteristics can be made. In contrast, for patients with pseudohypoparathyrodism type 1A (PHP1a), a genetic obesity syndrome caused by the loss of the maternal allele of the imprinted *GNAS* locus leading to disturbed MC4R signalling (5, 57, 58), decreased REE compared to multifactorial obesity (20, 59, 60), and compared to prediction equations has been described (21). In line with this, brain-specific *Gnas* knockout mice show reduced REE and increased feed efficacy (weight gain per kcal consumed) (58). Therefore, a decreased REE rather than hyperphagia is assumed to underlie the obesity associated with this syndrome. At present, REE measurements of 45 patients with PHP1a and 3 siblings with PHP1b have been described in literature (20, 21, 57, 59, 60), and both reduced (20, 21, 59) as well as normal (60) mREE adjusted for FFM compared to controls are reported in these studies. In our current study, we add REE data on 9 PHP1a and 2 PHP1b patients. Interestingly, we did not find evidence for a decreased REE except for one of our PHP1b patients with a REE% of 88.7%, even in our sensitivity analyses using only REE measurements in which an optimal steady state was achieved. Furthermore, mREE did not differ from children with multifactorial obesity after adjustment for FFM. Whether this arises from differing patient characteristics such as age, sex, and ethnic background, or REE and FFM measurement methods, remains to be investigated. Another possible explanation is that the specific gene variants in our patients and the previously described patients show differing residual *GNAS* activity *in vivo*. Our results regarding normal REE in PHP1a are in line with a recent report in patients with obesity caused by heterozygous pathogenic *GNAS* variants, where hyperphagia was reported for 11/22 patients and decreased REE compared to prediction equations were found in only 2/6 patients and were hypothesized to be associated with partial thyrotropin resistance (57). However, this effect can be excluded in our study as the PHP1a patients that had biochemical signs of hormone deficiencies were adequately supplemented at the time of the REE and body composition measurements. Together, our results suggest that the obesity phenotype of patients with PHP1a can be more variable than currently assumed and might not necessarily be driven by a decreased REE only.

### Hypothalamic Obesity

Our results confirm the decreased measured REE versus prediction equations in patients with hypothalamic obesity due to hypothalamic damage (24–26). The pathophysiologic mechanisms involved in these patients include reduced sympathetic tonus, thyroid metabolism, and brown fat activity as well as leptin and insulin resistance. Moreover, altered levels of α-MSH and satiety-regulating gut hormones can be seen, ultimately interfering with leptin-melanocortin signalling (61, 62). In previous studies, decreased mREE after adjustment for FFM compared to multifactorial obesity has been reported, namely in 18 children with hypothalamic obesity due to a hypothalamic lesion or damage (26), and in 8 patients with hypothalamic obesity after treatment for craniopharyngeoma (24). In contrast, other studies report a similar ratio of mREE per kg of FFM compared to controls, namely in 23 children after treatment for craniopharyngeoma (25) and 15 adults with various hypothalamic lesions (56). In our study, we did not find statistically significant differences in mREE adjusted for FFM between the patients with hypothalamic obesity compared to children with multifactorial obesity, although visual comparison of the regression fit lines (**Supplementary Figure S2**) shows a downward shift in hypothalamic obesity indicative of a lower mREE adjusted for FFM, in line with previous studies. The lack of statistical significance can probably be explained due to the small sample size of patients with hypothalamic obesity with available body composition measurements in our cohort. Altogether, our results suggest that their relatively low FFM (on average -11.7 kg compared to multifactorial obesity adjusted for age, sex, and BMI SDS) is an important driver of the lower REE compared to prediction equations in these patients. Interestingly, in two previous studies, the relationship between mREE and FFM was less strong or did not reach statistical significance in the subgroups of patients with hypothalamic obesity (24, 56). This suggests that, in contrast to multifactorial obesity, FFM might not be the most important factor determining REE in hypothalamic obesity. Another potential explanation for the differences between studies might be the different degrees and types of hypothalamic damage. As an example, our hypothalamic obesity group included two patients with meningomyelocele, both of which had a decreased REE% of 79.7% and 84.3%. This is in line with a recent study in 31 children with obesity with meningomyelocele where an average REE of 82% of predicted values was found (63). Importantly, a head-to-head comparison of these studies is hampered by the use of different methods to assess body composition (bioimpedance analysis [BIA] (25, 63) or dual energy x-ray absorptiometry [DXA] (24, 26, 56)) and different indirect calorimetry systems.

### Medication-Induced Obesity

We found that mREE in patients with medication-induced obesity is highly variable, yielding on average a slightly lower REE% of 95.5% and overestimation of +111 kcal/day versus predicted values. However, these differences were not statistically significant, probably due to the small sample size of this subgroup. The weight-inducing effects of most antipsychotic drugs, several antiepileptic drugs, and all corticosteroids are well-described (64, 65). Several mechanisms for inducing weight gain are proposed, such as central effects on the hypothalamus *via* leptin, neuropeptide Y (an orexigenic neuropeptide), serotonin, and adrenergic signalling (66–68). Although it can be hypothesized that these mechanisms could lead to a decreased REE, findings from clinical studies have not been consistent. In a prospective study of 54 adolescents who started a second-generation antipsychotic, mREE did not change after 1 year of treatment despite an average weight gain of +10.8kg, leading to a decrease in REE% (69). In contrast, other studies, e.g. in children on long-term treatment with valproic acid for epilepsy (70), did not detect differences in mREE adjusted for body weight versus healthy control children. For corticosteroids, the weight-inducing effects are most likely mediated through increased intake and central fat deposition (68), as both experimental administration of potent glucocorticoids as well as cortisol antagonists do not lead to altered REE (68, 71). Furthermore, REE adjusted for FFM is not altered in patients with Cushing’s syndrome, a disease characterized by highly elevated systemic cortisol levels (72). As the majority of our patients with medication-induced obesity used corticosteroids, this could explain the normal REE in this subgroup. Moreover, some of our patients with medication-induced obesity were not using this medication anymore at the time of REE measurement, which might explain the normal REE in this subgroup. Taken together, more research is needed to characterize the effects of weight-inducing medication on REE.

### Use of Prediction Equations in Children With Early-Onset Severe Obesity

Our main study finding was that a high variability in REE measurements compared to prediction equations were found across the entire study population. This is reflected by the large limits of agreement in our Bland-Altman analyses. An important reason for this variability is the inherent limitation of using REE prediction equations, which do not account for physiologic variability between patients with the same age, sex, and anthropometric characteristics that are used in the prediction equations. Other reasons for this variability might be related to patient characteristics such as variation in linear growth, pubertal stage, body composition (extremely low FFM), ethnic background, currently unidentified (poly)genetic risk factors affecting central energy expenditure regulation, or acute weight gain or loss, e.g. due to ongoing lifestyle interventions during REE measurement. Moreover, we cannot rule out that the lowered REE% in a subgroup of the patients with multifactorial obesity might be caused by underlying medical causes that we currently cannot diagnose with available techniques. We expected a high prevalence of decreased REE values in our study population based on the various underlying causes of our patients, but the Schofield equation on average predicted REE accurately in our population with a mean bias of only -12 kcal/day. The majority (56%) of our patients had a measured REE between 90-110% of predicted, and 21% and 24% of patients showed a decreased or elevated REE, respectively. In fact, the performance of the Schofield equation in our cohort was better than in most previous reported studies of pediatric patients with obesity. In these studies, higher mean biases and lower proportions of 21-61% of patients with predicted REE between 90-110% of measured REE were found (7, 16, 41–44, 73). An important drawback of the Schofield equations is that they are based on age categories (0-<3 years, 3-<10 years and 10-<18 years). Using the adjacent age category for patients at the limits of these categories would have explained the decreased REE of 1/60 patients and elevated REE of 12/69 patients. Thus, caution is warranted in the interpretation of the Schofield equations around the limits of the age categories, especially in case of elevated REE. To overcome this limitation of the Schofield equations, we performed sensitivity analyses using the Molnár equations. The largest external validation study to date recently showed that these have the highest ‘correct classification fraction’, that is, pREE within 90-110% of measured values, in Caucasian children with obesity (16). In these sensitivity analyses, we found similar results as in our analyses using the Schofield equations, which further strengthens our conclusions. It is important to realize that over the past years, several studies have investigated which prediction equations perform best in children with obesity. These studies show conflicting results varying from the Molnár equations (42), Schofield equations for height and weight (74, 75), Lazzer equations (43, 76), Mifflin equations (44), and WHO (77) equations. This variability might be related to different characteristics of the studied populations, such as age, sex, ethnic background and obesity severity, as well as differences in indirect calorimeters and test procedures and protocols. Hence, direct translation from any prediction equation into treatment advice in pediatric patients with severe obesity should be performed with caution. Additionally, measured REE should be related to body composition measures for correct interpretation. Our Bland-Altman analyses showed signs of proportionality of bias with increasing mean of mREE and pREE using both the Schofield (increasing underprediction) and Molnár (increasing overprediction) equations. Furthermore, sex differences were seen with regard to REE%, namely underprediction in girls relative to boys using the Schofield equations and overprediction using the Molnár equations. This should be taken into account when trying to interpret measured REE of older children and/or those with the most severe obesities.

### Implications for Clinical Practice

Our study underlines that measurement of REE can aid in developing a patient-tailored obesity approach in children with early-onset severe obesity. To estimate daily caloric needs in current clinical practice, TEE is calculated based on REE and child characteristics such as age, sex, and physical activity level (11–13, 16). Our results show that relying on predicted REE, whilst keeping all child characteristics such as physical activity level constant, would potentially overestimate or underestimate daily caloric needs by ≥10% in almost half of the children in our study population. As an example, this would translate into a significant average overestimation of daily caloric needs by 341 kcal/day in the 21% of patients with a decreased measured REE. Furthermore, specific therapeutic options can be considered in children with decreased measured REE, such as exercise training programs aimed at increasing or preserving lean body mass during weight loss (9). In adults, a recent non-randomized study showed that extensive phenotyping, including assessment of reduced energy expenditure, followed by a phenotype-tailored treatment approach, showed higher weight loss than standard-of-care treatment (78). Moreover, pharmacotherapy affecting central energy regulation can be considered in specific cases of children with severe obesity and reduced REE. Examples are dextroamphetamine or methylphenidate, which are centrally acting stimulants that increase serotonin, dopamine, and/or norepinephrine signalling. These drugs have shown promising results in smaller case series with non-syndromic genetic obesity and acquired hypothalamic obesity due to hypothalamic damage (79, 80). Furthermore, in patients with specific non-syndromic genetic obesity disorders such as POMC, LEPR and PCSK1 deficiency, the MC4R agonist setmelanotide has shown impressive results in terms of weight loss and increased satiety (81). This might be partially explained by increased energy expenditure (82). Finally, recent studies show favourable effects of glucagon-like peptide 1 (GLP-1) agonists, an anorexigenic gut hormone, both in adolescents with multifactorial obesity (83), as well as in adults with heterozygous *MC4R* variants and 16p11.2 deletion syndrome (84, 85). Whether this is mediated through changes in REE is currently unclear (85). Future studies should investigate whether children with severe obesity with decreased REE can benefit from these treatments.

### Strengths and Limitations

A major strength of our study is our relatively large cohort of patients with various rare, underlying medical disorders that lead to obesity. Our study expands knowledge of REE characteristics in hypothalamic obesity due to genetic disorders or hypothalamic damage. We are the first to describe REE and body composition characteristics in Temple syndrome and 16p11.2 deletion syndrome. Moreover, we describe REE characteristics of patients with all underlying medical causes that are described in current international pediatric obesity guidelines within one cohort (6). Another strength of our study is the standardized protocol in which all anthropometric, REE, and body composition measurements were collected. This was reflected by the fact that the sensitivity analysis using only REE measurements in which an optimal steady state was achieved showed similar numerical results as our main analyses. Furthermore, many studies that investigated REE in children with underlying medical causes of obesity only evaluated measured and predicted REE, and did not take body composition into account. By assessing body composition and comparing our results to children with multifactorial obesity, we could show that the differences between the patient subgroups disappeared when adjusting measured REE to FFM.

An inherent limitation of our study is that measured REE values were compared to predicted values. These are known to be inaccurate (7, 16, 42), despite being the only available external standard. We specifically chose to use the Schofield equations for our analyses based on the most recent systematic review (7), and performed additional sensitivity analyses using the Molnár equations based on the most recent and largest external validation study to date (16). This sensitivity analysis showed consistent outcomes, strengthening the generalizability of our study results. Furthermore, the use of 10% deviation from predicted values is an arbitrary cut-off, and we chose this cut-off because it is used in the large majority of studies comparing mREE with pREE (7, 16, 41–44, 73). Another limitation pertaining to the translation of our results into implications for clinical practice is that we did not measure physical activity level in this study. Ideally, a personalized dietary requirement advice would rely on direct measurement of TEE (using doubly labelled water) or measurement of REE (by indirect calorimetry) multiplied by an objectively measured physical activity level (by accelerometer). However, even if an objective estimate of TEE would have been achieved, compliance according to energy requirements is often an important issue to address during follow-up. It is important to realize that currently available techniques to diagnose and understand underlying medical causes of pediatric obesity have limitations, and some of our patients might have underlying polygenetic or epigenetic vulnerabilities or a combination of factors which we cannot currently classify into a separate subgroup of underlying medical cause. Moreover, as this study was performed in an academic obesity center, we cannot exclude the possibility that in a subgroup of our patients with multifactorial obesity, a singular underlying medical (e.g. genetic) cause might be present which we cannot detect with current knowledge and technologies. Notably, we measured body composition using air displacement plethysmography, which should be taken into account when comparing our results to studies that used BIA or DXA. As our study was cross-sectional, we cannot assess whether the decreased REE in our patients might have contributed to the development or clinical course of their obesity. Longitudinal studies investigating REE and TEE have scarcely been performed in children with multifactorial obesity (86, 87). These studies are yet to be performed in children with underlying medical causes of obesity to investigate the role of energy expenditure in the natural course of their obesity and response to treatment.

### Conclusion

In conclusion, we here show that resting energy expenditure in children with early-onset severe obesity due to multifactorial obesity or various underlying medical disorders that affect hypothalamic weight regulation demonstrates a large between-individual and between-disorder heterogeneity. A substantial number of patients have decreased or elevated values compared to prediction equations, corresponding to underprediction or overprediction of daily caloric needs of hundreds of calories. In half of our population, body composition data were available. Subgroup analyses in this group showed that children with hypothalamic obesity had a significantly lower measured REE than predicted and a lower FFM, whereas children with non-syndromic genetic obesity showed a significantly higher measured REE than predicted and a higher FFM. No differences in measured REE were found after adjustment for FFM between the patients with vs. without underlying medical causes. Thus, our study underlines the importance of measuring REE and body composition in children with early-onset severe obesity with or without underlying medical causes that affect hypothalamic weight regulation. This knowledge can aid in developing patient-tailored treatment approaches, such as personalized dietary interventions or physical activity interventions aimed at increasing lean body mass. Furthermore, pharmacologic treatment affecting central energy expenditure regulation could be considered in children with decreased measured REE.

## Data Availability Statement

The datasets presented in this article are not readily available because they contain information that may compromise participants’ anonymity, but can be made available upon reasonable request. Requests to access the datasets should be directed to the CGG Steering Committee (Prof. Erica L.T. van den Akker, centrumgezondgewicht@erasmusmc.nl).

## Ethics Statement

The studies involving human participants were reviewed and approved by the Medical Ethics Committee of the Erasmus MC, Rotterdam, The Netherlands. Written informed consent to participate in this study was provided by the participants’ legal guardian/next of kin.

## Author Contributions

OA, EK: conceptualisation, data curation, formal analysis, investigation, methodology, project administration, validation, visualisation, writing – original draft, verifying the underlying data. MW: data curation, formal analysis, investigation, methodology, project administration, validation, visualisation, writing – review & editing. SB: data curation, investigation, methodology, project administration, validation, writing – review & editing, verifying the underlying data. ER, MH: conceptualisation, investigation, methodology, resources, supervision, validation, visualisation, writing – review & editing. BV, CG: conceptualisation, data curation, formal analysis, investigation, methodology, project administration, supervision, validation, visualisation, writing – review & editing, verifying the underlying data. EA: conceptualisation, data curation, formal analysis, investigation, methodology, project administration, resources, software, supervision, validation, visualisation, writing – review & editing, verifying the underlying data. All authors contributed to the article and approved the submitted version.

## Funding

This work was supported by the Elisabeth Foundation (grant name ObesEcare), a non-profit foundation supporting academic research.

## Conflict of Interests

The authors declare that the research was conducted in the absence of any commercial or financial relationships that could be construed as a potential conflict of interest.

## Publisher’s Note

All claims expressed in this article are solely those of the authors and do not necessarily represent those of their affiliated organizations, or those of the publisher, the editors and the reviewers. Any product that may be evaluated in this article, or claim that may be made by its manufacturer, is not guaranteed or endorsed by the publisher.
