# Supplementary material for: Resting Energy Expenditure and Body Composition in Children and Adolescents With Genetic, Hypothalamic, Medication-Induced or Multifactorial Severe Obesity
Source: Front Endocrinol (Lausanne). 2022 Jul 11;13:862817. doi: 10.3389/fendo.2022.862817 (PMC9309560; doi:10.3389/fendo.2022.862817)
Supplement: Supplementary file 1 [file DataSheet_1.docx]

Supplementary Material

**Supplementary figure captions**

Supplementary Figure 1. Scatter plots showing the relations between measured REE and FFM, and REE% and age and BMI SDS. The dots represent the individual patients. The line represents the linear regression fit line across the study population
Abbreviations: (m)REE, (measured) resting energy expenditure; FFM, fat-free mass; kcal, kilocalories; SDS, standard deviation score.

Supplementary Figure S2. Scatter plots showing the relations between measured REE and FFM stratified on underlying medical causes. No differences were found between the intercept nor slope for each the underlying medical causes compared to multifactorial obesity. The dots represent the individual patients. The line represents the linear regression fit line for each underlying medical cause: non-syndromic genetic obesity: REE = 28.9*FFM + 697.9, R^2^=0.78; syndromic genetic obesity: REE = 25.3*FFM + 660.4, R^2^=0.79; hypothalamic obesity: REE = 25.5*FFM + 520.5, R^2^=0.95; medication-induced obesity: REE = 29.6*FFM + 594.4, R^2^=1.00; multifactorial obesity: REE = 25.7*FFM + 739.3.9, R^2^=0.71.
Abbreviations: REE, resting energy expenditure; FFM, fat-free mass; kcal, kilocalories; SDS, standard deviation score.

Supplementary Figure S3. Measured REE expressed as percentage of predicted REE (by Schofield equations) stratified on sex. Male patients with non-syndromic genetic obesity had higher REE% compared to children with multifactorial obesity (p<0.05) whereas both male as well as female children with hypothalamic obesity had lower REE% (p<0.05 and p <0.01, respectively). The dots represent the individual patients. The bars represent the mean + standard error of the mean. The light green shaded area indicates a REE% between 90 and 110%.
Abbreviations: REE, resting energy expenditure.

Supplementary Figure S4. Measured REE expressed as percentage of predicted REE stratified on ethnicity. Dutch patients with non-syndromic genetic obesity had higher REE% compared to children with multifactorial obesity (p<0.05) whereas Dutch children with hypothalamic obesity had lower REE% (p<0.05 and p <0.01, respectively). The dots represent the individual patients. The bars represent the mean + standard error of the mean. The light green shaded area indicates a REE% between 90 and 110%.
Abbreviations: REE, resting energy expenditure.

Supplementary Figure S5. Bland-Altman plot for the agreement between mREE and pREE_Molnar_ (by Molnár equations). The dots represent the individual patients. The middle dashed line represents the mean absolute (A) or relative bias (B) across the study population. The upper and lower dashed lines represent the upper and lower limits of agreement (mean bias ± 1.96 SD) of mREE and pREE_Molnár_. The solid line represents the linear regression fit line.
Abbreviations: mREE, measured resting energy expenditure; pREE, predicted resting energy expenditure (using the Molnár equations).

Supplementary Figure S6. Bland-Altman plot for the agreement between mREE and pREE_Lazzer_ (by Lazzer equations). The dots represent the individual patients. The middle dashed line represents the mean absolute (A) or relative bias (B) across the study population. The upper and lower dashed lines represent the upper and lower limits of agreement (mean bias ± 1.96 SD) of mREE and pREE_Lazzer_. The solid line represents the linear regression fit line.
Abbreviations: mREE, measured resting energy expenditure; pREE, predicted resting energy expenditure (using the Lazzer equations).

**Supplementary Tables**

*Supplementary Table S1. Comparison of baseline characteristics between patients with and without BOD POD measurement.*

|  | **No BOD POD measurement  (n=146)** | **BOD POD measurement available (n=146)** | **P-value** |
| --- | --- | --- | --- |
| Age, years | 10.1 (4.4) | 11.6 (4.0) | 0.002 |
| Sex, female, n (%) | 92 (63) | 80 (55) | 0.15 |
| Ethnicity, Dutch, n (%) | 100 (69) | 102 (70) | 0.48 |
| Height, cm | 143.0 (24.8) | 151.9 (21.1) | 0.001 |
| Height SDS | 0.37 (1.46) | 0.29 (1.31) | 0.63 |
| Weight, kg | 67.0 (33.1) | 78.1 (32.5) | 0.004 |
| Weight SDS | 3.64 (1.70) | 3.77 (1.34) | 0.50 |
| BMI, kg/m^2^ | 30.4 (7.8) | 32.0 (6.9) | 0.07 |
| BMI SDS | 3.73 (1.20) | 3.78 (0.92) | 0.70 |
| Abbreviations: BMI, body mass index; SDS, standard deviation score. Data presented as mean (SD), unless otherwise stated. | | | |

*Supplementary Table S2. Correlation coefficients between REE and patient characteristics.*

| **Parameter** | **Correlation with** | **All patients (n=292)** | **Non-syndromic genetic obesity (n=29)** | **Syndromic genetic obesity (n=28)** | **Hypothalamic obesity (n=10)** | **Medication-induced obesity (n=7)** | **Multifactorial**  **obesity (n=218)** |
| --- | --- | --- | --- | --- | --- | --- | --- |
| REE% | Age | -0.06 | -0.12 | 0.11 | 0.02 | - | -0.04 |
| REE% | BMI SDS | -0.09 | -0.21 | 0.06 | -0.47 | - | -0.13* |
| mREE | FFM^a^ | 0.85^a^*** | .79^a^*** | 0.77^a^*** | - | - | 0.84^a^*** |
|  |  |  |  |  |  |  |  |
|  |  |  |  |  |  |  |  |
| Abbreviations: mREE, measured resting energy expenditure; REE%, ratio mREE/predicted REE (based on Schofield equations); FFM, fat-free mass; SDS, standard deviation score; -, correlation not assessed due to small sample size. The presented correlation coefficients are Pearson’s *r* (in case of n ≥ 25) or Kendall’s τ (in case of n between 10 and 25).  ^a^ Available for n=146 patients with available BOD POD measurement (18 non-syndromic, 13 syndromic, 5 hypothalamic, 2 medication-induced, and 108 multifactorial obesities) * P<0.05 ** P<0.01 *** P<0.001 | | | | | | | |

*Supplementary Table S3. Sensitivity analysis of REE and body composition characteristics including only REE measurements in which an optimal steady state was achieved*

|  | **All patients (n=172)** | **Non-syndromic genetic obesity (n=17)** | **Syndromic genetic obesity (n=12)** | **Hypothalamic obesity**  **(n=5)** | **Medication-induced obesity (n=3)** | **Multifactorial**  **obesity (n=135)** |
| --- | --- | --- | --- | --- | --- | --- |
| mREE, kcal/day | 1752 (468) | 1904 (495) | 1517 (313) | 1507 (287) | 1854 (396) | 1760 (477) |
| pREE, kcal/day | 1748 (501) | 1799 (494) | 1527 (326) | 1666 (330) | 1949 (300) | 1760 (521) |
| Mean bias (mREE – pREE), kcal/day | 4 (241) | 106 (238) | -10 (142) | -159 (261) | -94.8 (165) | 0 (247) |
| REE% | 101.3 (12.7) | 106.7 (13.7) | 99.6 (7.4) | 91.4 (15.0) | 94.8 (8.2) | 101.3 (12.7) |
| Lowered mREE, n (%) | 32 (19) | 3 (18) | 1 (8) | **3 (60)*** | 1 (33) | 24 (18) |
| Elevated mREE, n (%) | 47 (27) | 7 (41) | **0 (0)*** | 1 (20) | 0 (0) | 39 (29) |
| FFM, %BW | 55.1 (8.4)^a^ | 56.3 (5.8)^a^ | 59.8 (8.1)^a^ | 47.0 (15.6)^a^ | 56.2 (10.3)^a^ | 54.7 (8.3)*^a^* |
| Abbreviations: mREE, measured resting energy expenditure; pREE, predicted resting energy expenditure (based on Schofield equations); REE%, ratio mREE/pREE; FFM, fat-free mass; %BW, percentage of body weight; kcal, kilocalories. Data presented as mean (SD), unless otherwise stated. ^a^ Available for n=103 patients with available BOD POD measurement (13 non-syndromic, 8 syndromic, 3 hypothalamic, 2 medication-induced, and 77 multifactorial obesities) * P<0.05 vs multifactorial obesity. | | | | | | |

*Supplementary Table S4. Sensitivity analysis of REE characteristics using the Lazzer equations to calculate predicted REE in the subgroup of measurements in which an optimal steady-state was achieved (n=103 [71%] of patients in whom measured body composition data were available)*

|  | **All patients (n=103)** | **Non-syndromic genetic obesity (n=13)** | **Syndromic genetic obesity (n=8)** | **Hypothalamic obesity**  **(n=3)** | **Medication-induced obesity (n=2)** | **Multifactorial**  **obesity (n=77)** |
| --- | --- | --- | --- | --- | --- | --- |
| mREE, kcal/day | 1797 (476) | 1908 (489) | 1541 (284) | 1369 (289) | 1997 (438) | 1816 (486) |
| pREE_Molnár_, kcal/day | 1805 (415) | 1839 (468) | 1619 (298) | 1562 (257) | 2018 (146) | 1823 (422) |
| Mean bias (mREE – pREE_Lazzer_), kcal/day | -8 (211) | 70 (145) | -79 (182) | -193 (36) | -21 (293) | -6 (222) |
| REE% | 99.3 (12.0) | 103.9 (8.7) | 95.5 (10.9) | 87.2 (4.2) | 98.4 (14.6) | 99.4 (12.0) |
| Lowered mREE, n (%) | 22 (21) | 0 (0) | 2 (25) | 2 (67) | 1 (50) | 17 (22) |
| Elevated mREE, n (%) | 17 (17) | 4 (31) | 0 (0) | 0 (0) | 0 (0) | 13 (17) |
| Abbreviations: mREE, measured resting energy expenditure; pREE_Lazzer_, predicted resting energy expenditure (based on Lazzer equations); REE%, ratio mREE/ pREE_Lazzer_; kcal, kilocalories.  Data presented as mean (SD), unless otherwise stated. No statistically significant differences were observed in pairwise comparisons for each of the underlying medical causes compared to multifactorial obesity (all P>0.05) | | | | | | |

*Supplementary Table S5. Sensitivity analysis of REE characteristics using the Molnár equations to calculate predicted REE*

|  | **All patients (n=292)** | **Non-syndromic genetic obesity (n=29)** | **Syndromic genetic obesity (n=28)** | **Hypothalamic obesity**  **(n=10)** | **Medication-induced obesity (n=7)** | **Multifactorial**  **obesity (n=218)** |
| --- | --- | --- | --- | --- | --- | --- |
| mREE, kcal/day | 1705 (491) | 1884 (612) | **1479 (360)*** | 1535 (236) | 1710 (342) | 1719 (490) |
| pREE_Molnár_, kcal/day | 1623 (430) | 1753 (517) | **1423 (316)*** | 1651 (241) | 1654 (296) | 1629 (434) |
| Mean bias (mREE – pREE_Molnár_), kcal/day | 83 (209) | 131 (228) | 56 (143) | **-116 (201)**** | 55 (293) | 89 (207) |
| REE% | 105.1 (13.6) | 107.0 (13.8) | 104 (9.3) | **93.5 (12.3)**** | 104.1 (16.1) | 105.6 (13.9) |
| Lowered mREE, n (%) | 36 (12) | 3 (10) | 3 (11) | **4 (40)**** | 1 (14) | 25 (12) |
| Elevated mREE, n (%) | 108 (37) | 11 (38) | 7 (25) | 2 (20) | 4 (57) | 84 (39) |
| Abbreviations: mREE, measured resting energy expenditure; pREE_Molnár_, predicted resting energy expenditure (based on Molnár equations); REE%, ratio mREE/pREE_Molnár_; kcal, kilocalories.  Data presented as mean (SD), unless otherwise stated.  * P<0.05 ** P<0.01 vs multifactorial obesity. | | | | | | |

*Supplementary Table S6. Sensitivity analysis of REE characteristics using the Lazzer equations to calculate predicted REE*

|  | **All patients (n=146)** | **Non-syndromic genetic obesity (n=18)** | **Syndromic genetic obesity (n=13)** | **Hypothalamic obesity**  **(n=5)** | **Medication-induced obesity (n=2)** | **Multifactorial**  **obesity (n=108)** |
| --- | --- | --- | --- | --- | --- | --- |
| mREE, kcal/day | 1801 (481) | 1973 (519) | **1518 (370)*** | 1446 (234) | 1997 (438) | 1819 (480) |
| pREE_Molnár_, kcal/day | 1822 (416) | 1872 (441) | 1643 (312) | 1669 (252) | 2018 (146) | 1838 (429) |
| Mean bias (mREE – pREE_Lazzer_), kcal/day | -21 (213) | **101 (184)*** | -125 (191) | **-223 (72)*** | -21 (293) | -20 (212) |
| REE% | 98.5 (12.1) | **105.0 (9.4)*** | 91.9 (12.9) | **86.6 (3.7)*** | 98.4 (14.6) | 98.7 (12.0) |
| Lowered mREE, n (%) | 33 (23) | **0 (0)*** | 4 (31) | **4 (80)*** | 1 (50) | 24 (22) |
| Elevated mREE, n (%) | 20 (14) | **6 (33)*** | 0 (0) | 0 (0) | 0 (0) | 14 (13) |
| Abbreviations: mREE, measured resting energy expenditure; pREE_Lazzer_, predicted resting energy expenditure (based on Lazzer equations); REE%, ratio mREE/ pREE_Lazzer_; kcal, kilocalories.  Data presented as mean (SD), unless otherwise stated.  * P<0.05 ** P<0.01 vs multifactorial obesity. | | | | | | |
