# Supplementary figures and images for: Resting Energy Expenditure and Body Composition in Children and Adolescents With Genetic, Hypothalamic, Medication-Induced or Multifactorial Severe Obesity
Source: Front Endocrinol (Lausanne). 2022 Jul 11;13:862817. doi: 10.3389/fendo.2022.862817 (PMC9309560; doi:10.3389/fendo.2022.862817)

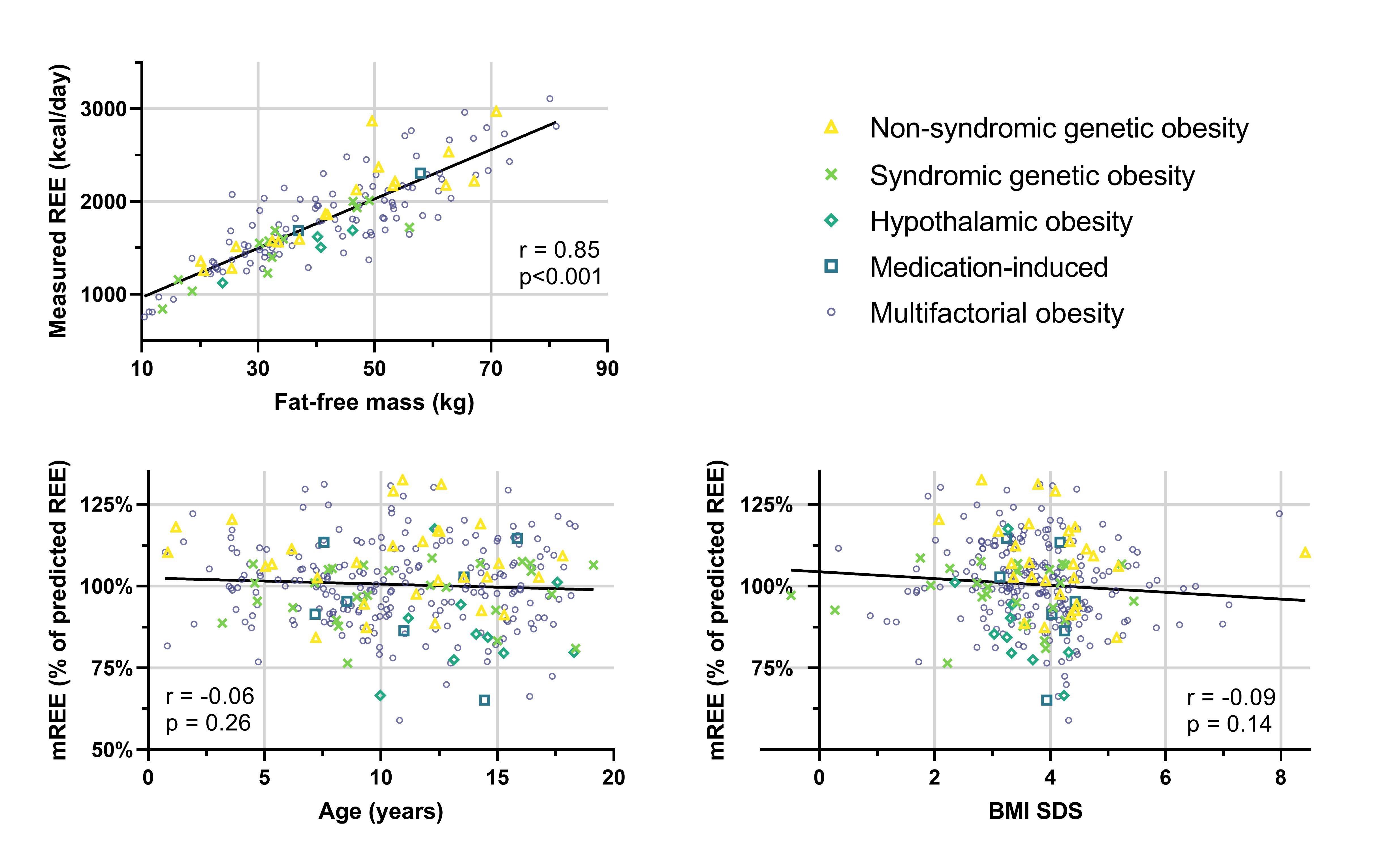

Supplement: Supplementary file 2 [file Image_1.tif]

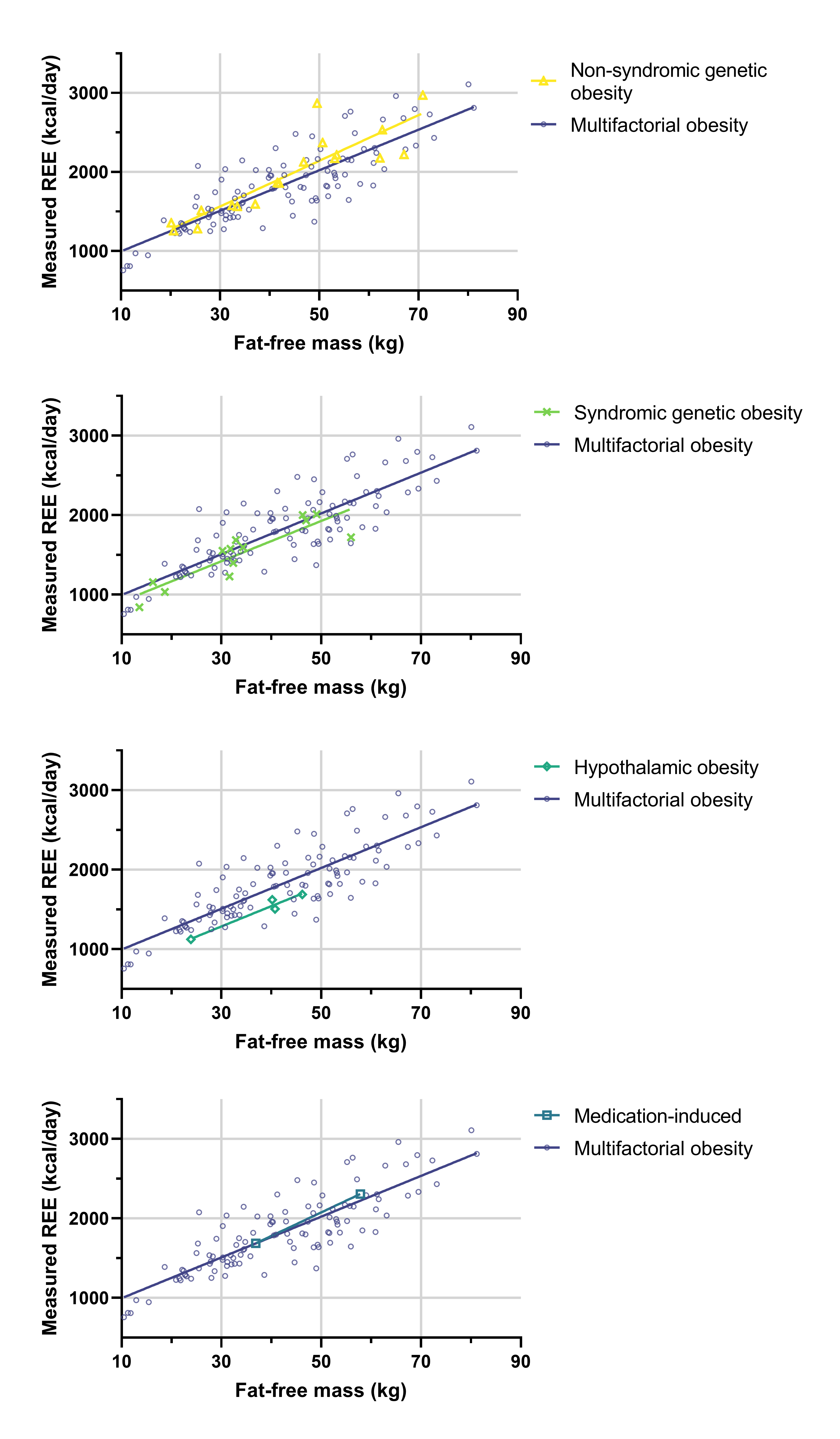

Supplement: Supplementary file 3 [file Image_2.tif]

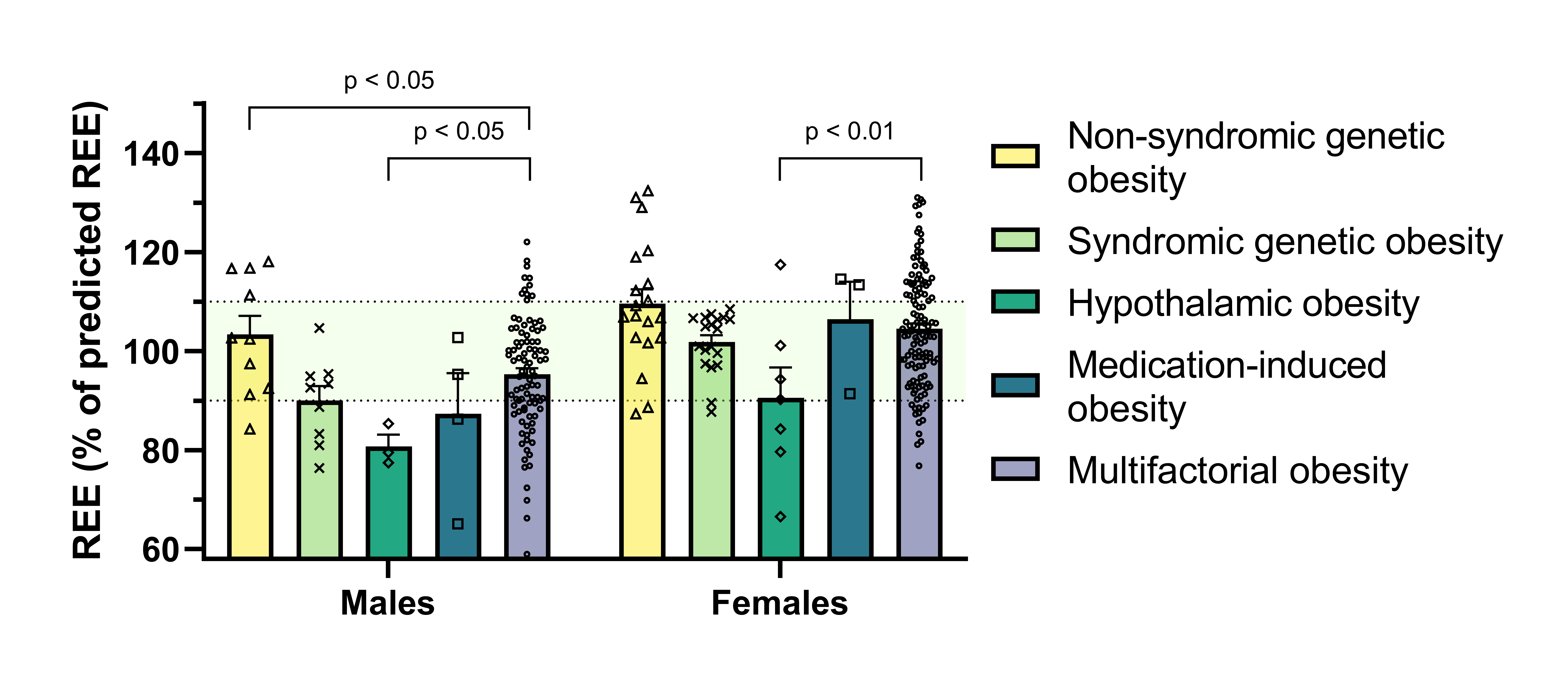

Supplement: Supplementary file 4 [file Image_3.tif]

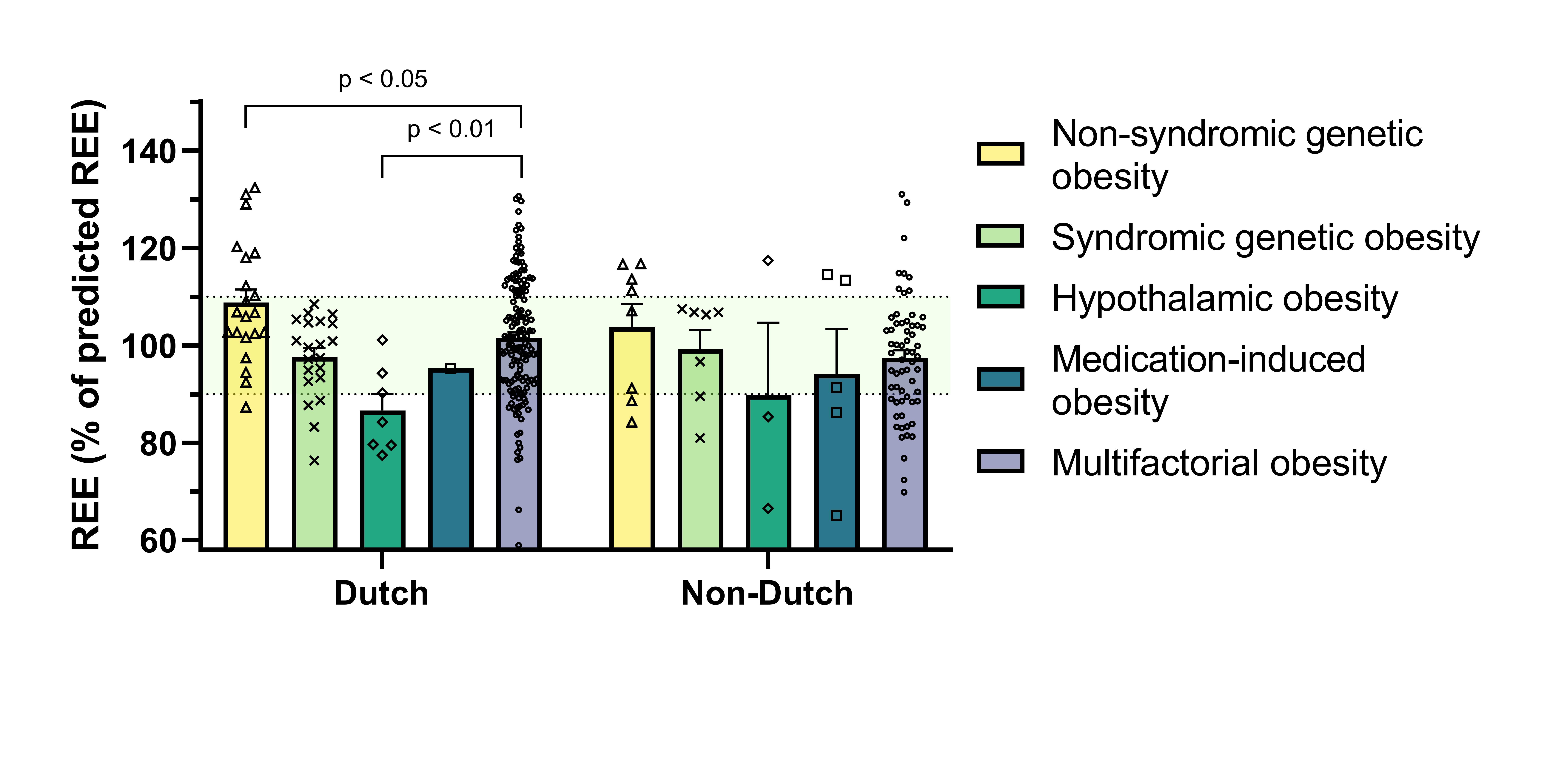

Supplement: Supplementary file 5 [file Image_4.tif]

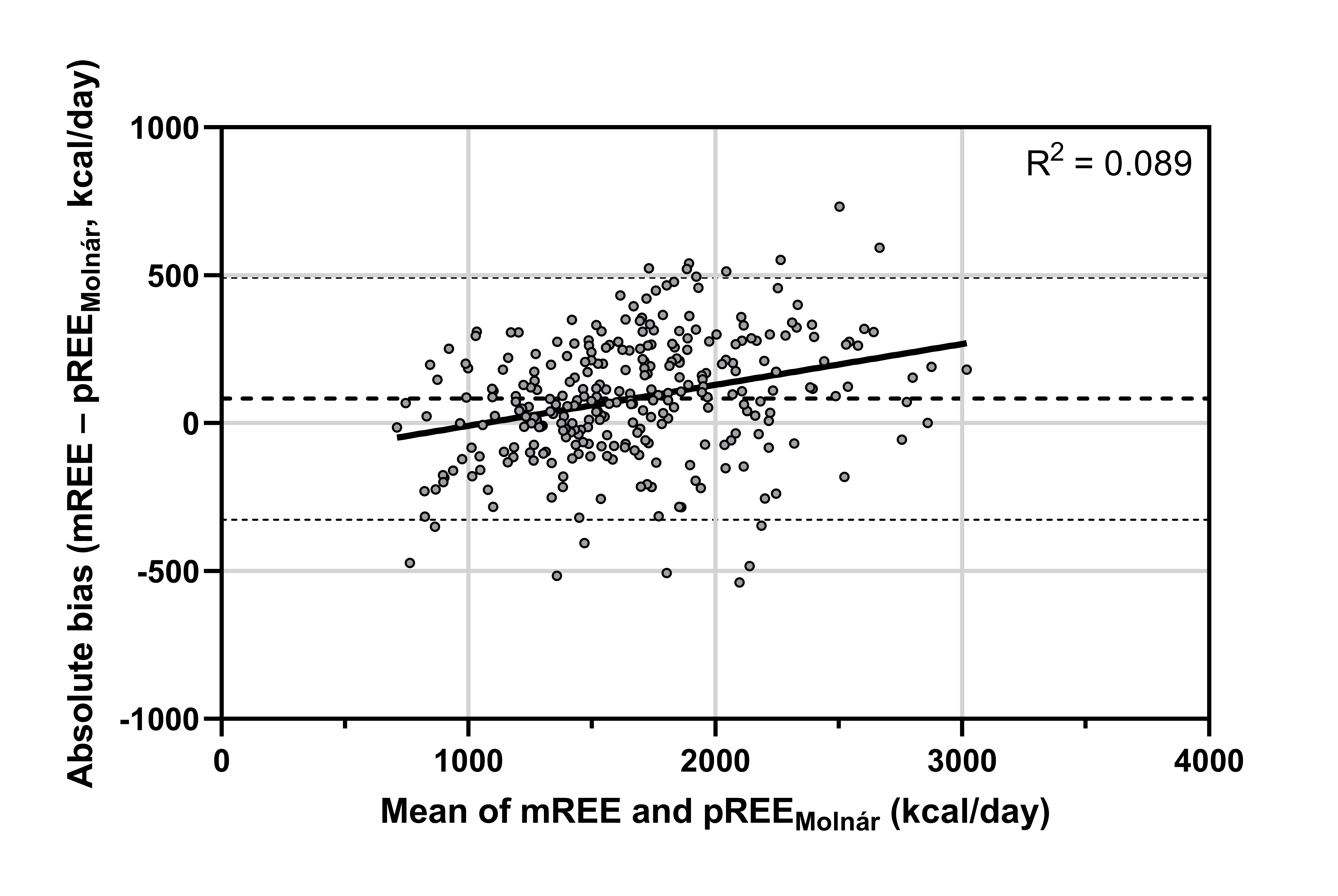

Supplement: Supplementary file 6 [file Image_5.tif]

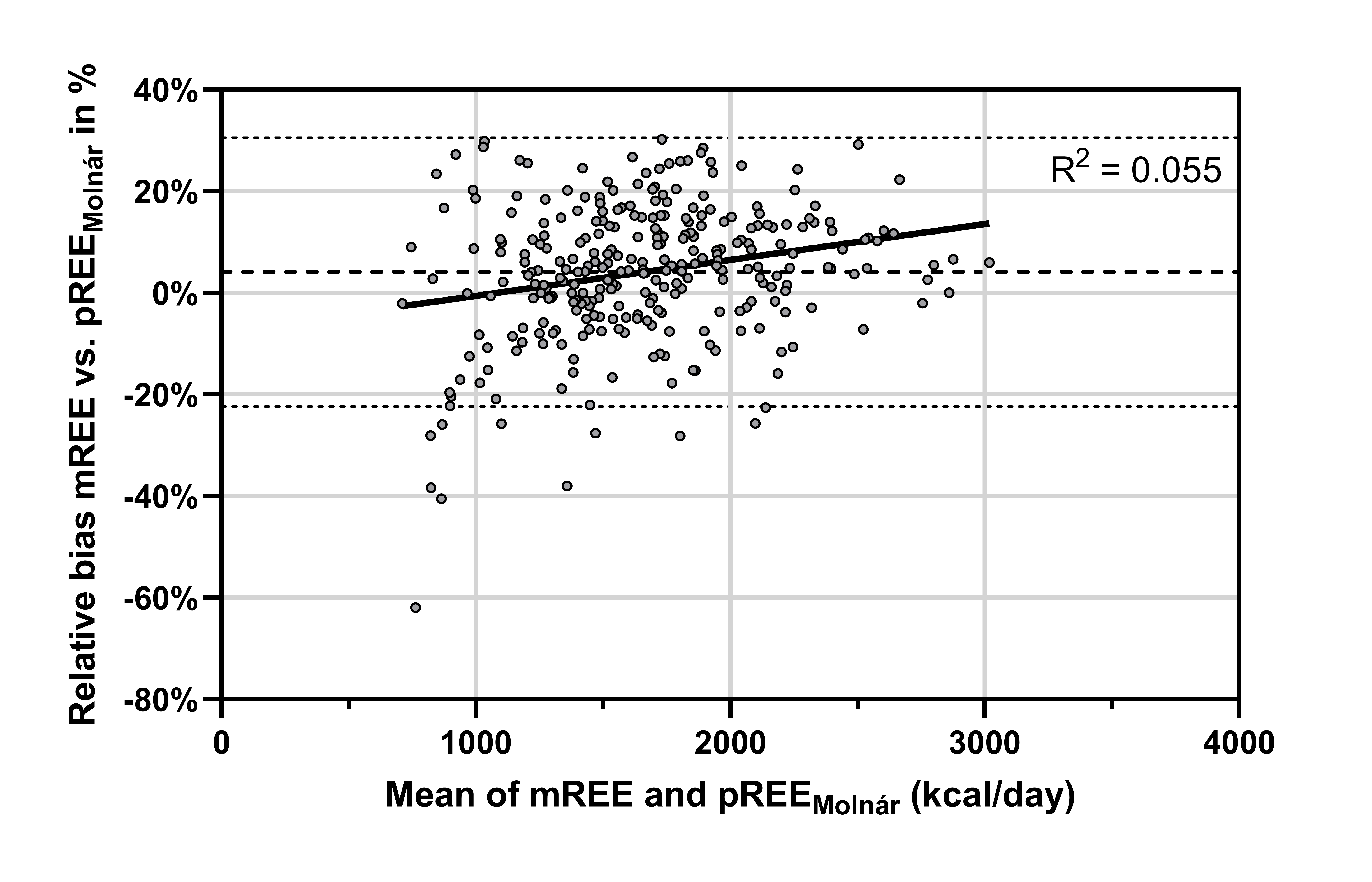

Supplement: Supplementary file 7 [file Image_6.tif]

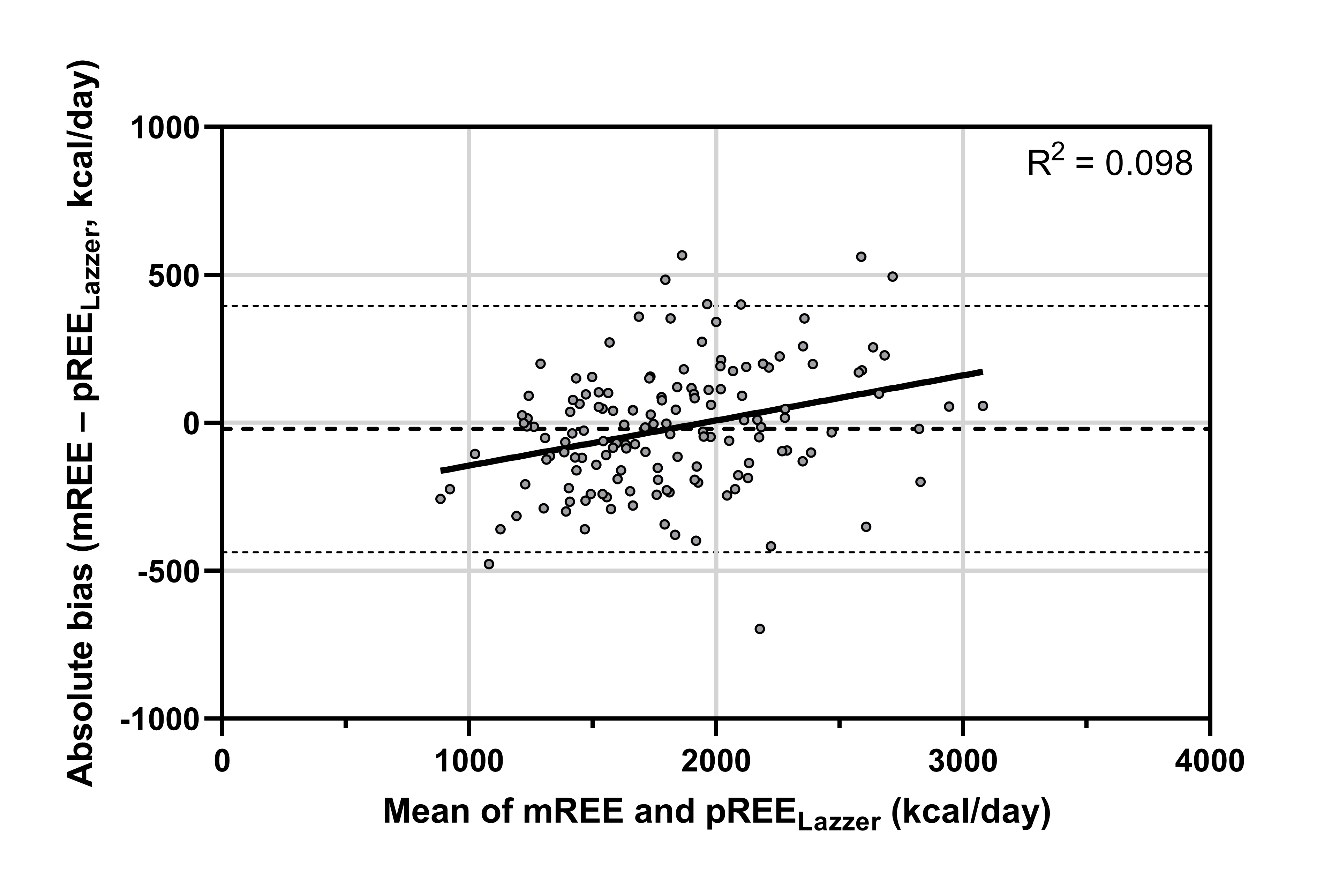

Supplement: Supplementary file 8 [file Image_7.tif]

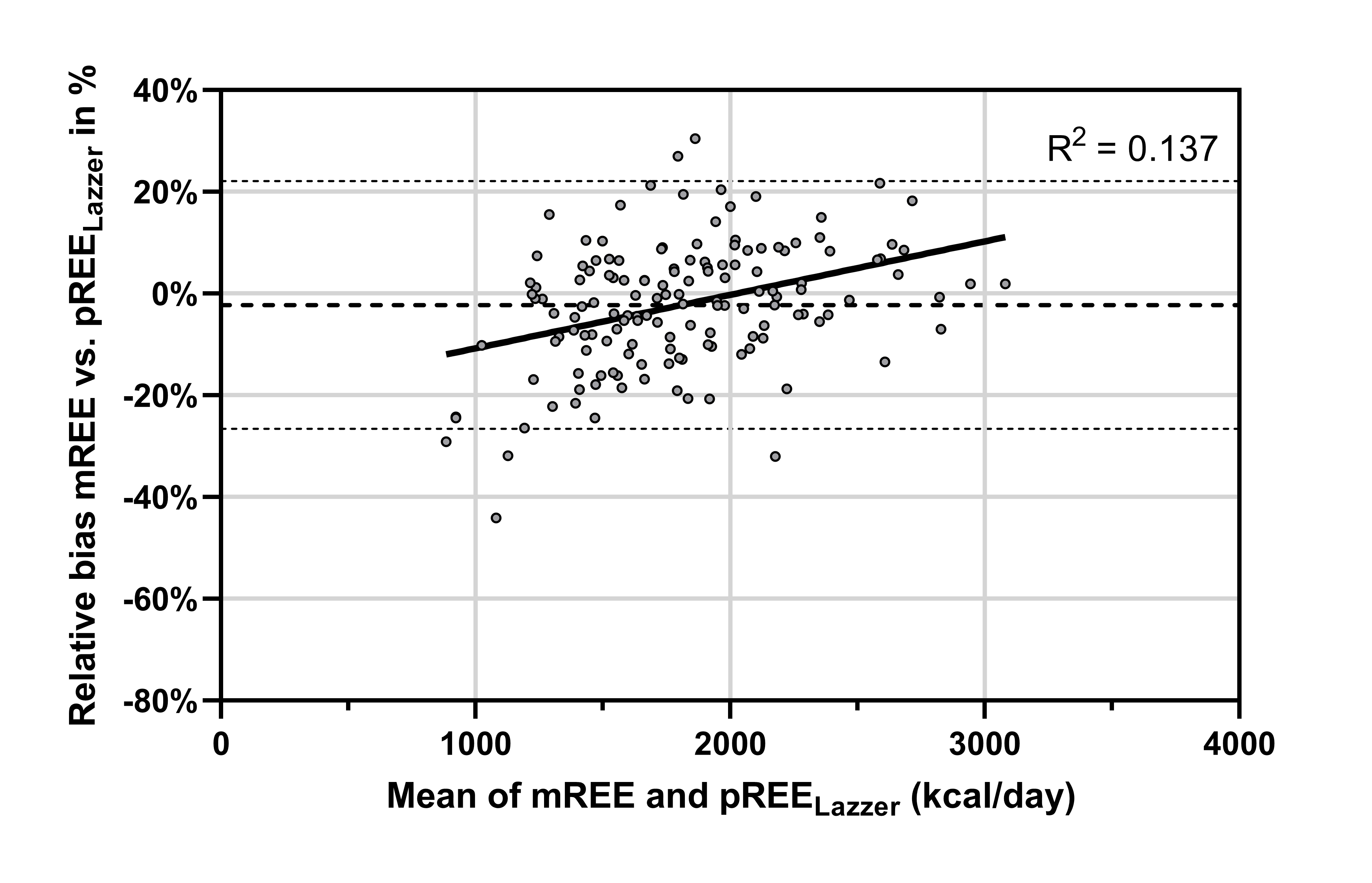

Supplement: Supplementary file 9 [file Image_8.tif]
